# High TGF-β signature predicts immunotherapy resistance in gynecologic cancer patients treated with immune checkpoint inhibition

**DOI:** 10.1038/s41698-021-00242-8

**Published:** 2021-12-17

**Authors:** Ying Ni, Ahmed Soliman, Amy Joehlin-Price, Peter G. Rose, Anda Vlad, Robert P. Edwards, Haider Mahdi

**Affiliations:** 1 Center for Immunotherapy and Precision Immuno-Oncology, Lerner Research Institute, Cleveland Clinic, 9500 Euclid Avenue, Cleveland, OH 44195 USA; 2 Department of Pediatrics, Case Western University, 2103 Cornell Rd, Cleveland, OH 44106 USA; 3 Department of Anatomic Pathology, Pathology and Laboratory Medicine Institute, Cleveland Clinic, 9500 Euclid Avenue, Cleveland, OH 44195 USA; 4 Section of Gynecologic Oncology, Women’s Health Institute, Cleveland Clinic, 9500 Euclid Avenue, Cleveland, OH 44195 USA; 5 Magee Women’s Hospital, University of Pittsburgh Medical Center, Pittsburgh, PA 15213 USA; 6 Magee Women’s Research Institute, University of Pittsburgh, Pittsburgh, PA 15213 USA

**Keywords:** Immunotherapy, Cancer genomics

## Abstract

Various immune signatures predictive of resistance to immune checkpoint inhibitors (ICI) have been described in multiple solid cancers, but still under-investigated in gynecological (GYN) cancer. For 49 GYN cancer patients included in our study, without transcriptome signature, immune-related toxicity was the only clinical predictor of ICI treatment response (*p* = 0.008). The objective clinical response was the only predictor of progression-free survival (ICI-PFS, *p* = 0.0008) and overall survival (ICI-OS, *p* = 0.01). Commonly used ICI marker PD-L1 expression negatively correlated with progression-free survival (ICI-PFS) (*p* = 0.0019). We performed transcriptome and signaling pathway enrichment analyses based on ICI treatment responses and the survival outcome, and further estimated immune cell abundance using 547 gene markers. Our data revealed that TGF-β regulated signaling pathway was noted to play an important role in immunotherapy failure. Using our 6-genes TGF-β score, we observed longer ICI-PFS associated with lower TGF-β score (8.1 vs. 2.8 months, *p* = 0.046), which was especially more prominent in ovarian cancer (ICI-PFS 16.6 vs. 2.65 months, *p* = 0.0012). Further, abundant immunosuppressive cells like T-regulatory cells, eosinophils, and M2 macrophages were associated with shorter ICI-OS and correlated positively with *CD274* and *CTLA4* expressions. This study provides insight on the potential role of TGF-β in mediating immunotherapy resistance and cross-talking to immunosuppressive environment in GYN cancer. The TGF-β score, if validated in a larger cohort, can identify patients who likely to fail ICI and benefit from targeting this pathway to enhance the response to ICI.

## Introduction

Immunotherapy with immune checkpoint inhibitors (ICI) has emerged as a promising option in other solid tumors like lung and urothelial cancers and melanoma. In gynecologic cancers, the ICI response rates range from 11–17% in the recurrent setting^1–4^. Patients with deficiency in their DNA mismatch repair system have been shown to respond well to PD-1 inhibitors with reported response rates between 24–57% in recurrent settings^5,6^. However, these patients account for only 20–30% of all endometrial cancer cases and represent a significantly smaller (less than 10%) fraction in other gynecologic cancers. In microsatellite stable endometrial cancer, the response rate to ICI was only 6–13%^2,7^. Similarly in ovarian cancer, the response rate to ICI was low, ranging from 11–15% in platinum-resistant, recurrent settings^8,9^. PD-L1 expression has been associated with response to ICI in some cancers like lung cancer. However, the predictive role of PD-L1 expression in gynecologic cancers is controversial with mutually conflicting results. Increasing evidence points to intratumor immune cell infiltration, especially by activated CD8 T cells, as a reliable predictor of increased survival^10^. Furthermore, tumors with increased tumor mutational burden (TMB) display neoantigens, are T-cell inflamed, and respond better to ICI^11^. Recently, an 18-gene T-cell inflamed gene expression profile signature was identified in pretreatment tumor samples, as a predictor of response to pembrolizumab in melanoma, gastric as well as head and neck cancers^12^. However, if this signature also applies to gynecologic cancers remains unclear. TMB has been shown to correlate positively with response to anti-PD1 therapy. Recently, the FDA approved pembrolizumab in the second line setting in patients with TMB >10 mutations / megabase (mut/Mb)^11^. Nevertheless, the optimal cutoff for TMB remains controversial, and it does not take into consideration immunosuppressive factors that may contribute to resistance to immunotherapy within the tumor immune microenvironment (TME). Therefore, we need to better understand mechanisms driving resistance to immune checkpoint inhibition and identify a subset of patients who will better benefits from immunotherapy. It is also critically important to establish and validate predictive biomarkers that help identify immunosuppressive factors that can be targeted in a rational combination immunotherapy approach. These biomarkers provide a significant value in personalized immunotherapy approach and need to be validated in future large studies including prospective clinical trials to confirm their predictive role.

Focusing on patients with recurrent gynecologic cancer, we sought to perform a comprehensive transcriptomic analysis of the TME and to identify factors could interact with TME and potentially predict ICI treatment response or resistance in GYN cancer.

## Results

### Immune-related toxicities correlate with response whereas response to anti-PD-1/PD-L1 immunotherapy correlates with survival outcome after starting ICI

To comprehensively assess the correlation between gene expression profile and immune response, we collected tumor samples from a retrospective series of 49 patients diagnosed with gynecology cancer (included endometrial, cervical, or ovarian cancers) who were treated with immune checkpoint inhibitors such as nivolumab or pembrolizumab. Of all 49 patients, 19 (39%) had an objective response (either partial or complete response by RECIST 1.1). Baseline patient characteristics and available survival and clinical data of our patients are summarized in Table 1.

**Table 1:** Clinico-pathological summaries of 49 gynecology patients included in this study.

Characteristic	*N* = 49^a^
DiseaseSite	
Ovarian	14 (29%)
Endometrial	27 (55%)
Cervical	8 (16%)
StageAtDiagnosis	
I	13 (29%)
II	2 (4.4%)
III	17 (38%)
IV	13 (29%)
NA	4
MSI	
Stable	16 (33%)
High	17 (35%)
NA	16 (33%)
Age	68 (62, 74)
NA	1
Histology	
Adenocarcinoma	1 (2.1%)
Clear cell	5 (11%)
Endometrioid	16 (34%)
Mixed endometrioid and clear cell	1 (2.1%)
Serous	17 (36%)
Squamous	7 (15%)
Unknown	2
Cycles	8 (3, 11)
PriorChemotherapyLines	
NA	1 (2.0%)
0	4 (8.2%)
1	19 (39%)
2	6 (12%)
3	9 (18%)
4	5 (10%)
5	1 (2.0%)
7	2 (4.1%)
8	1 (2.0%)
11	1 (2.0%)
PlannedRegimen	
AVELUMAB	2 (4.1%)
NIVOLUMAB	19 (39%)
PEMBROLIZUMAB	28 (57%)
Toxicity	23 (48%)
NA	1
Response	
Progressive disease	21 (45%)
Responded disease	20 (43%)
Stable disease	6 (13%)
NA	2
Death	29 (60%)
NA	1
Overall Survival (months)	13 (6, 21)
NA	1
Progression	33 (72%)
NA	3
Progression-Free Survival (months)	6 (3, 12)
NA	2

^a^Statistics presented: *n* (%); Median (IQR).

**Fig. 1:**
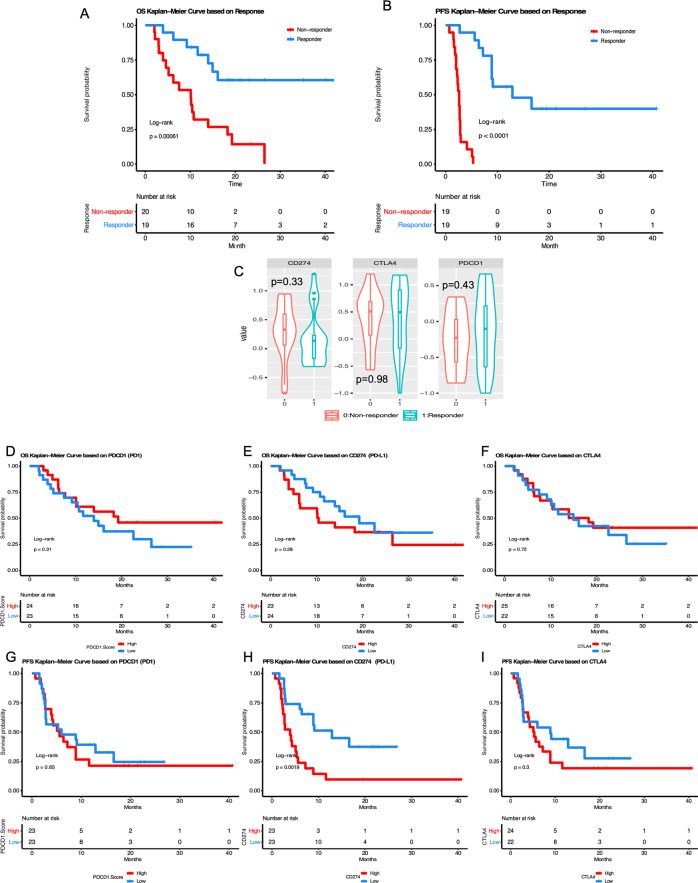
Survival curve for an entire cohort based on response to immunotherapy and major know molecular biomarkers *CD274* (*PD-L1)* and *CTLA4* expression. **A** Kaplan–Meier curves of overall survival (OS) based on response to immunotherapy patient received; **B** Kaplan–Meier curves of Progression-free survival (PFS) based on response to immunotherapy patient received; **C** Violin plot of *CD274 (PD-L1)*, *CTLA4, and PDCD1 (PD1)* expression between responders and non-responders; **D**–**F** Kaplan–Meier curves of OS based on *PDCD1, CD274* (*PD-L1), and CTLA4* expression; **G**–**I** Kaplan–Meier curves of PFS based on *PDCD1, CD274* (*PD-L1), and CTLA4* expression; For panels **D**–**G**, marker group “High” or “low” was defined by median expression of each marker gene.

**Fig. 2:**
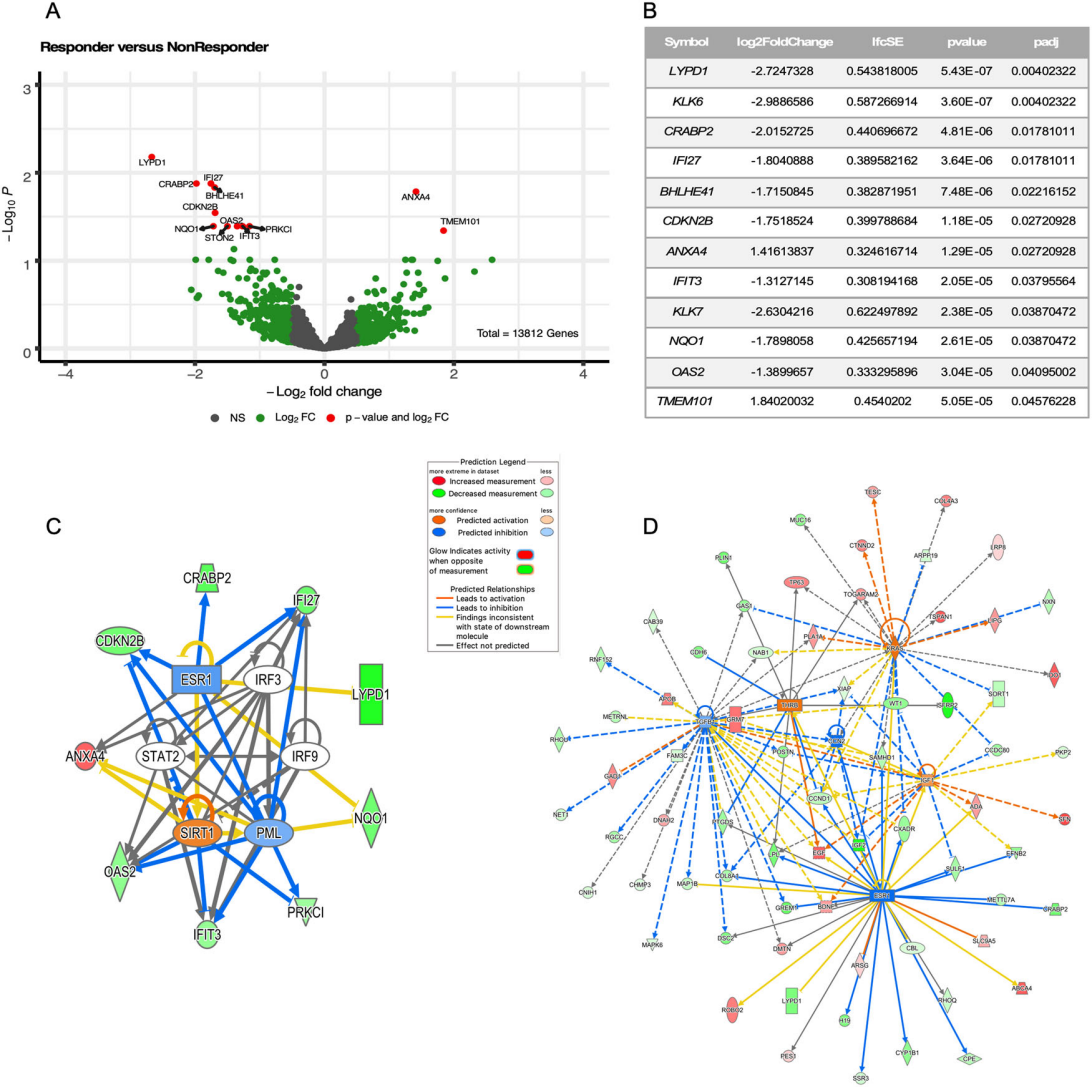
Differential expression profile in 49 gynecological cancer patients based on responses to immunotherapy. **A** Volcano plot of genome-wide differential expression analysis, with *x*-axis as -Log2 (Fold Change), *y*-axis as -Log10 (adjusted *p* value). Green dots highlighted genes with fold change ≥ 2, red dots highlighted genes with fold change equal or larger than 2, and adjusted *p* value ≤ 0.05, gray dots presented as genes were not significantly expressed between responders and non-responders. **B** The list of 12 significantly expressed genes between responders and non-responders, with corresponding log2(folder change), log2(folder change) standard error, *p* value attained by the Wald test, adjusted *p* value corrected for multiple testing using Benjamini and Hochberg method. **C** IPA network of top upstream regulators, based on the 12 significantly expressed genes among the entire cohort. **D** IPA network of 6 top common upstream regulators (*KRAS, ESR1, IGF1, CCN2, THRB*, and *TGFB1*) derived from individual disease analysis.

When we evaluated all demographic and clinical characteristics including disease site, response pattern, age at treatment initiation, stage, MSI status, and immune-related toxicities in a multivariable regression analysis, response to immune checkpoint inhibition was found to be only positively correlated with immune-related toxicity (*p* = 0.008, Table 2). Furthermore, based on multi-variable survival analysis, response to immune checkpoint inhibition was found to be the only factor significantly associated with overall and progression-free survival (from initial of immunotherapy treatment) (*p* = 0.010 and 0.0008 respectively, Table 3). The lack of significance between immune-related toxicity and outcome is likely related to its significant correlation/interaction with a response. Responding patients (R) who had an objective response (partial or complete response) to immune checkpoint inhibition had a median overall survival (OS) of 17.2 months compared to the 10.1 months of non-responding patients (NR) (*p* < 0.001, log-rank test), as well as a median progression-free survival (PFS) of 12.9 months in R compared to the 2.6 months in NR patients (*p* < 0.001, log-rank test, Figs. 1 and 2).

**Table 2:** Association of clinical features with IO response outcome, based on general linear regression model.

	Estimate	Std. error	*t* value	Pr(>|t|)
(Intercept)	1.406165	0.513224	2.74	0.01096*
DiseaseSite1OV2Endo3Cerv2	0.07354	0.245623	0.299	0.76701
DiseaseSite1OV2Endo3Cerv3	−0.023845	0.243281	−0.098	0.92267
Stage2	−0.520145	0.419982	−1.238	0.2266
Stage3	−0.425539	0.222825	−1.91	0.06726^#^
Stage4	−0.373122	0.243768	−1.531	0.13794
MSI1S2U3NA2	0.043776	0.244521	0.179	0.8593
MSI1S2U3NA3	−0.093975	0.211345	−0.445	0.66025
Age	−0.013399	0.007415	−1.807	0.08236^#^
Toxicity (yes)	0.514275	0.177901	2.891	0.00766**

Signif. codes: **0.001 < *p* < 0.01; *0.01 < *p* < 0.05; ^#^0.05 < *p* < 0.1.

**Table 3:** Association of clinical features with IO survival outcome, based on Cox proportional hazard model.

	OS		PFS	
	exp(coef)	Pr(>|z|)	exp(coef)	Pr(>|z|)
Resp1resp0prog	0.17865	0.0106*	0.024631	0.000795***
DiseaseSite1OV2Endo3Cerv2	0.30678	0.1159	0.820203	0.741241
DiseaseSite1OV2Endo3Cerv3	0.38097	0.2344	0.826595	0.785085
Stage2	1.40738	0.8008	0.54165	0.638433
Stage3	1.01989	0.9796	1.171952	0.83355
Stage4	0.64347	0.5676	1.7394	0.492262
MSI1S2U3NA2	1.8205	0.4286	2.431407	0.261984
MSI1S2U3NA3	1.54989	0.5038	1.252915	0.661159
Age	1.0355	0.1851	1.007193	0.742769
Toxicity1	0.90657	0.8813	0.313008	0.088383^#^

Signif. codes: ***p < 0.001; *0.01 < *p* < 0.05; ^#^0.05 < *p* < 0.1.

### PDL1 expression correlates negatively with progression-free survival on ICI

Tumor expression of immune checkpoint molecules like PD1, PD-L1 (which binds to PD-1), and CTLA4 (a T-cell immune suppressant that counteracts the stimulatory activity of CD28) are biomarkers of interest to correlate with response to ICI. We investigated if *PD1 (PDCD1)*, *PD-L1(CD274)*, or *CTLA4* mRNA expression are correlated with response and survival outcome (PFS and OS) after initiating ICI therapy in our cohort. No correlation was noted between *PD1*, *PD-L1* or *CTLA4* and clinical response (*p* = 0.43, 0.33 and 0.98 respectively, Fig. 1C) or OS (*p* = 0.31, 0.28 and 0.72 respectively, Fig. 1D–F) but high *PD-L1* expression significantly correlated with lower PFS (*p* = 0.0019, Fig. 1H). In contrast, *PD1* and *CTLA4* expression did not correlate with PFS (*p* = 0.65 and 0.30, Fig. 1G, I).

### Genome-wide DEGs analysis identifies 12 genes signature in response to immunotherapy outcome

Genome-wide gene expression data including 13812 genes passed quality control for all patients. We identified 12 genes that were significantly differentially expressed in R compared to NR patients across the three cancer types (Fig. 2A), comprising 10 downregulated genes and 2 upregulated genes (*ANXA4, TMEM101*, Fig. 2B). Using Ingenuity Pathway Analysis (IPA, Qiagen, Redwood City, CA), we found among the upstream regulators of these 12 genes are important interferon regulatory factors (IRF3 and IRF9), along with PML and SIRT1 genes (Fig. 2C).

### Major up-regulators were analyzed in different disease sub-cohorts

To highlight the specific signaling pathways particular to the cancer location, differential gene expression analysis was also performed for each cancer type (ovarian, cervical, and endometroid) individually. In contrast to few genes expressed differently in endometrial cancer patients who responded to immunotherapy versus non-responders, there are 116 differentially expressed genes (DEGs) in cervical cancer patients and 312 genes in ovarian cancer patients stratified by responders and non-responders to immunotherapy (Supplementary Table 1, p.adjust <0.05, log2FC >1). Canonical pathways curated based on differentially expressed genes using IPA are listed in Supplementary Table 2.

Consistent with the analysis performed with the entire cohort, immune signaling showed up as a key regulator in both cervical and ovarian cancers. Specifically, *TGFB1, WNT3A*, and their targets are the top signaling pathway inhibited in immunotherapy responded cervical cancer. *KRAS* and *IGF1* are the top activated signaling, along with *WNT1* inhibited, in immunotherapy responded ovarian cancer. Among all predicted signaling up-regulators for ovarian and cervical cohorts, there are 6 molecules noted in both datasets: *KRAS, ESR1, IGF1, CCN2, THRB*, and *TGFB1* (Fig. 2D), and interesting to note, all these molecules are all inter-connected.

### TGF-β signaling pathway genes expression correlated with survival benefit while on immunotherapy

**Fig. 3:**
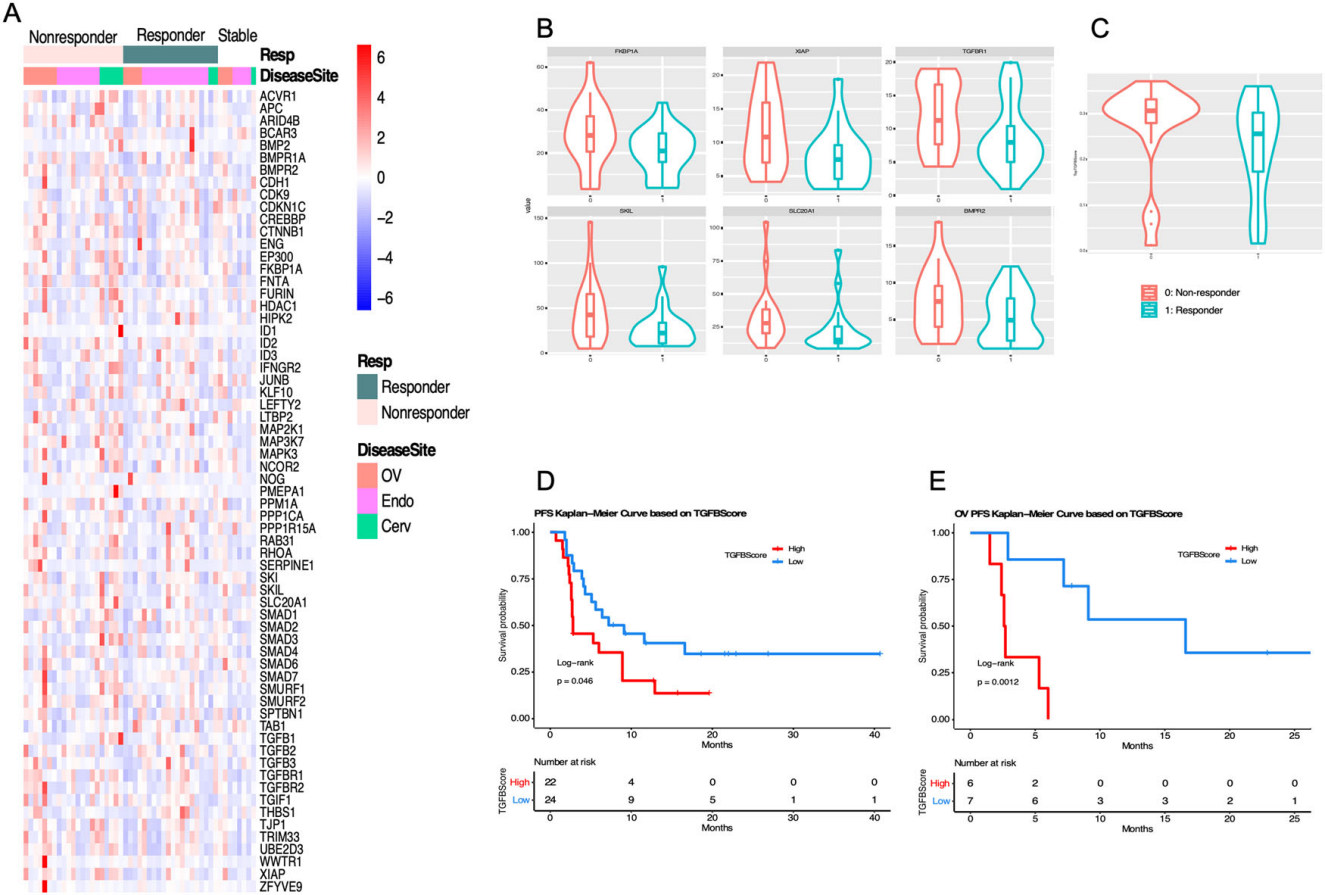
TGF-β signaling pathway gene expression profile and survival curve based on TGF-β score generated. **A** Heatmap of all 65 curated TGF-β pathway genes, sorted by responders and non-responders in each 3 disease types; **B** Violin plot of top 6 individual genes (*SLC20A1, XIAP, TGFBR1, BMPR2, FKBP1A*, and *SKIL*) expression in TGF-β pathway between responders and non-responders; **C** Violin plot of the TGF-β score generated using these 6 genes between responders and non-responders; **D** Kaplan–Meier curves of PFS based on TGF-β score in the entire cohort; **E** Kaplan–Meier curves of PFS based on TGF-β score in ovarian cancer patients. For panels **D** and **E**, the TGF-β score group “High” or “low” was defined by the median expression of the TGF-β score.

Given that not only TGF-β interacted with all other 5 molecules based on the above agnostic analysis, but also the potential clinical application of TGF-β inhibitors in cancer immunotherapy combination treatment, we investigated the role of TGF-β signaling pathway in immunotherapy treatment responses in our cohort. We first curated 65 genes regulated by TGF-β from multiple pathway databases and compared their expression levels among responders versus non-responders (Fig. 3A). Univariate analysis showed that 6 genes (*SLC20A1, XIAP, TGFBR1, BMPR2, FKBP1A*, and *SKIL*) had lower expression in patients who responded to immunotherapy (*p* < 0.1, Supplementary Table 3, Fig. 3B). We then generated a TGF-β score by applying single-sample enrichment analysis^13^ as described in the Method section, to use for subsequent analysis. Patients with low TGF-β scores had significantly improved PFS while on immunotherapy (Fig. 3C, 8.15 months vs 2.8 months, *p* = 0.046), which was most noticeable in ovarian cancer despite the smaller sample size (16.6 months vs 2.65 months, *p* = 0.0012, Fig. 3D and Supplementary Fig. 1). There was a trend toward improved overall survival in the entire cohort and the ovarian subtype (Supplementary Fig. 1).

To validate the association between TGF-β score and survival we observed in our gynecology cancer patients, we tested our 6-gene signature using the online tool TIDE: Tumor Immune Dysfunction and Exclusion as described in Method. Interestingly, compared to other existing biomarkers, our TGF-β score showed good predictive value (AUC > 0.7) in 2 studies (Nathanson 2017_CTLA4_Melanoma_Pre and Miao2018_ICB_Kidney_Clear, Supplementary Fig. 2A) and a significant negative association with available overall survival in 2 studies (Liu2019_PD1_Melanoma_Ipi and Braun2020_PD1_Kidney_Clear, Supplementary Fig. 2B, C).

### Immune cells landscape within tumor immune microenvironment of gynecological tumors who received immune checkpoint inhibition

**Fig. 4:**
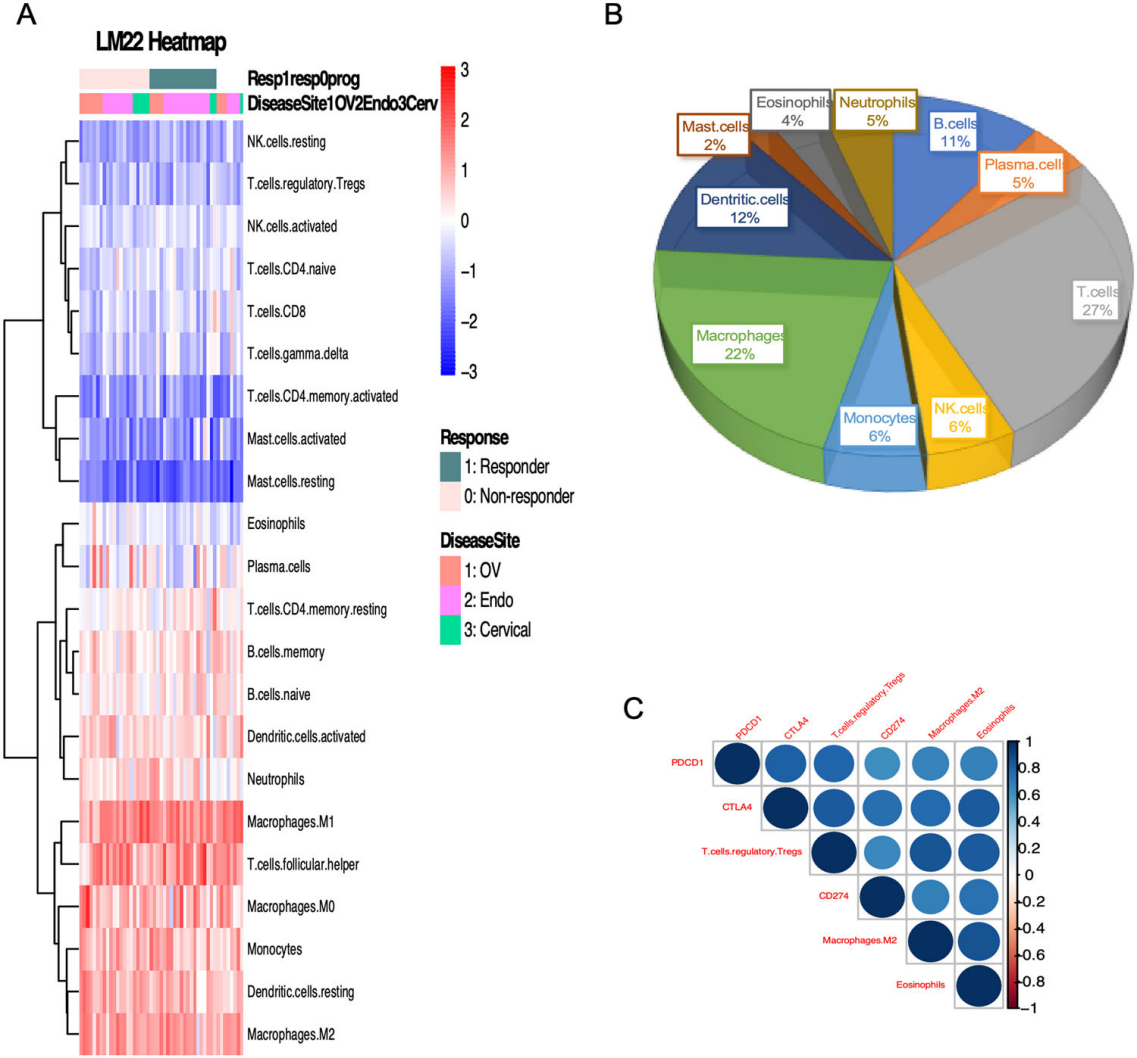
Expression of different immune cell abundance. **A** Heatmap of all 22 immune cell abundance and status (LM22) in the entire cohort, sorted by responders and non-responders in each 3 disease types, scaled within each sample. **B** Pie chart of the prevalence of each immune cell abundance among all samples. **C** Correlation plot among marker genes *PDCD1 (PD1)*, *CD274* (PD-L1), *CTLA4* and immune cells M2 macrophage, eosinophils, and regulatory T cells.

Based on the observation that the molecular signaling alteration in response to immunotherapy is enriched in immune signaling, we decided to look into the overall profile of the immune microenvironment using our transcriptome data. We characterized 22 immune composition subsets in silico using our bulk RNAseq data, by extracting genes representing these immune cells and activating status^14^. We calculated the cell type signature score (LM22 score) based on the representing genes in each functional annotation in Fig. 4A (Heatmap of LM22) (as referenced). Using this bioinformatic estimation, compared to T cells, NK cells, and Mast cells, all patients had relatively higher expression of genes related to macrophage-monocyte, B cells, as well as dendritic cells (Fig. 4B). Neutrophils, eosinophils, and plasma cells were also prominent. No difference was noted in the composition of immune cells when stratified by the response, MSI status, or cancer site (Supplementary Fig. 3).

### T-regulatory, M2 macrophages and eosinophils (known major source of TGF-β) were predictive of worse overall survivals while on immunotherapy

To access the predictive value of immune cell abundance to predict survival outcome, we performed multivariable Cox regression analysis in our LM22 score dataset while adjusting for age, stage, and disease site as co-variants. Among all 22 cell types and activation states, higher expression of CD4 naïve T cells, follicular helper and regulatory T cells, resting NK cells, M2 macrophage, resting mast cells, and eosinophils are associated with worse overall survival. Whereas CD4 memory resting T cells, gamma/delta T cells, and activated mast cells were associated with better overall survival. For progression-free survival, follicular helper T cells, resting NK cells, and eosinophils were associated with worse survival; resting and activated memory CD4 T cells showed association with better survival (Table 4).

**Table 4:** Multi-variate analysis for OS and PFS with LM22 in entire cohort, with age, clinical stage, and disease site as co-variates.

	OS			PFS	
	coef	Pr(>|z|)		coef	Pr(>|z|)
Plasma.cells	7.74E+01	4.58E−02*	T.cells.CD4.memory.resting	−1.20E+02	1.55E−02*
T.cells.CD4.naive	2.40E+02	1.35E−02*	T.cells.CD4.memory.activated	−4.15E+01	1.87E−02*
T.cells.CD4.memory.resting	−1.91E+02	8.74E−03**	T.cells.follicular.helper	1.06E+02	5.68E−04***
T.cells.follicular.helper	2.34E+02	1.88E−04***	NK.cells.resting	4.36E+01	2.12E−02*
T.cells.regulatory.Tregs	1.18E+02	1.30E−02*	Eosinophils	6.61E+01	3.46E−03**
T.cells.gamma.delta	−5.10E+02	1.97E−03**	Age	4.36E−02	3.28E−01
NK.cells.resting	2.07E+02	1.10E−03**	Stage2	−3.80E−01	8.94E−01
NK.cells.activated	−9.11E+01	4.98E−02*	Stage3	3.58E+00	5.34E−03**
Macrophages.M2	1.52E+02	8.25E−03**	Stage4	3.89E+00	2.64E−02*
Mast.cells.resting	2.83E+02	2.04E−03**	DiseaseSite_Endo	2.09E+00	0.041474*
Mast.cells.activated	−2.55E+02	1.18E−03**	DiseaseSite_Cerv	2.39E+00	0.289849
Eosinophils	3.51E+02	1.15E−03**			
Age	4.54E−01	7.47E−04***			
StageII	6.61E+00	1.31E−01			
StageIII	5.95E+00	8.91E−03**			
StageIV	−5.50E−01	8.67E−01			
DiseaseSite_Endo	−7.18E+00	0.019077*			
DiseaseSite_Cerv	−3.02E+01	0.008344**			

Signif. codes: ***p < 0.001; **0.001 < *p* < 0.01; *0.01 < *p* < 0.05; ^#^0.05 < *p* < 0.1.

Then we sought to assess the correlation of the immunosuppressive immune cells that were significantly associated with worse OS or PFS with PD-L1 and CTLA4 expression. We noted positive correlation between T regulatory cells, eosinophils, M2 macrophages with *CD274* (correlation efficiency 0.64, 0.73, 0.68, respectively) and *CTLA4* (correlation efficiency 0.83, 0.83, 0.76, respectively) expression respectively (Fig. 4C).

## Discussion

The role of PD-L1 expression in predicting immunotherapy benefit has been controversial in gynecologic cancer with mutually controversial results. Interestingly, using mRNA expression, we observed that high PD-L1 expression correlated with poor progression-free survival on ICI. This can be explained by several mechanisms. First PD-L1 expression is a proxy of immunosuppressive immune non-T cells like T-regulatory cells, M2 macrophages, MDSCs, and others. Therefore, high PD-L1 expression could potentially be correlated with the high abundance of these immune cells reflecting the immunosuppressive environment. We sought to assess such correlation and noted a positive correlation between PD-L1 expression and these immunosuppressive cells. Another explanation is that PD-L1 also represents a prognostic factor. This has been reported by Hamanishi et al., where the authors demonstrated the correlation between high PD-L1 and lower survival as well as lower tumor-infiltrating T cells in ovarian cancer^15^.

We demonstrated that the TGF-β signaling pathway was among the top regulator genes and is interacting with WNT, KRAS, and IGF pathways contributing to the prediction of immunotherapy response. The 6-genes TGF-β signature we created showed positive correlation with immunotherapy resistance, which is most prominent in the ovarian cancer subtype despite the small sample size. We showed that myeloid cells are very prominent in the TME and correlated positively with the worse outcome while on immunotherapy. Of particular interest, we described the role of M2 macrophages, T-regulatory cells, and eosinophils which are known to produce TGF-β within the tumor immune microenvironment. The immunosuppressive role of eosinophils has not been described before in these cancers in the setting of immunotherapy which we hypothesize to be mediated by the TGF-β immunosuppressive effect.

TGF-β has emerged as a critical mediator of cancer therapy resistance, including ICI resistance^16–24^. Other studies have shown that TGF-β attenuates the response to ICI and contributes to immune exclusion and evasion in some solid tumors including bladder, colon, and esophageal squamous cell cancers^23–28^. However, the data on the role of TGF-β pathway in mediating resistance to immunotherapy with ICI in gynecologic cancer in general and especially ovarian cancer is limited. TGF-β signaling has been shown to provide a favorable TME immune-mediated tumor clearance by T cells and NK cells. For example, TGF-β suppresses the function of cytotoxic T cells (CTL), while disruption of TGF-β signaling enhances CD8+ T-cell-mediated and NK cell-mediated anti-tumor immune responses^29–31^. In addition to the suppression of T-cell and NK cell function, TGF-β also has a significant impact on the myeloid cell lineages including recruiting and promoting M2 TAMs. These cells will eventually compete with dendritic cells and suppress antigen presentation. Further, TGF-β can suppress the activation, maturation, and differentiation of macrophages, dendritic cells, and neutrophils, which weakens the innate immune system. Therefore, the suppressed innate immune system will negatively affect the adaptive anti-tumor immune response and allow cancer cells to escape the immune response. TGF-β has been shown to induce polarization of TAMs toward the M2 phenotype via up-regulation of SNAIL pathway^32^. Therefore, high TGF-β within the TME can block the development of M1 TAMs and induce the formation and activation of the M2 immunosuppressive phenotype. TGF-β is also produced by M2 TAMs and myeloid-derived suppressor cells (MDSC) and plays a significant role in inducing the expression of genes that are involved in activating the M2 macrophage phenotype^33^. These data are consistent with our findings of the negative correlation of M2 macrophages with survival outcome while on immunotherapy in our cohort. This might indicate a potential dynamic interaction of TGF-β with tumor-associated macrophages to promote M2 phenotype which eventually further feeds into the TGF-β cycle. These data shed the light on the role of TGF-β in mediating resistance to ICI in gynecologic cancer which has not been shown before. Further, we were able to validate our 6-genes signature in two other publicly available datasets.

Other differentially expressed pathways noted on our analyses were KRAS, IGF, WNT, and IFN pathways, all of which interact with the TGF-β pathway. Grasso et al showed that T-cells infiltration and IFN-γ signaling signature were associated with increased likelihood of response to immune checkpoint therapy in melanoma^34^. Activation of the STING pathway can induce an innate immune response and tumor rejection mediated by phosphorylation of IRF3 and inducing strong production of IFN-α/β^35^. TGF-β could therefore suppress IFN-α and β pathways leading to suppression of the innate immune response limiting tumor regression mediated by this pathway. Further, TGF-β inhibition was found to restore the production of IFN-α by activated MHCII tumor-associated macrophages and enables tumor regression by STING pathway activation^35^.

Similarly, the RAS/RAF/MAPK pathway has been investigated and correlated with response to immunotherapy with immune checkpoint inhibition^36–38^. KRAS pathway has been shown to interact with the TGF-β pathway. In one study, oncogenic RAS and TGF-β signaling pathways have been shown to correlate with each other and the cross-interaction is mediated by neuropilin-1 (NRP1), a receptor that can interact with multiple growth factors including TGF-β for promoting tumorigenesis and immune suppression^36^. Other mechanisms described include phosphorylation of SMAD by kinases controlled by the RAS pathway, which ultimately control the translocation and activation of SMAD proteins^39^. RAS activation phosphorylates smad2/3 through ERK/MAPK pathway leading to blockade of their nuclear transcription and thereby eventually genes transcriptions^40^. Lastly, both TGF-β and RAS pathways regulate gene transcriptions^39^.

Another interesting finding in our study is the role of eosinophils. Eosinophils are a type of granulated white blood cells, circulating in the blood and marginating into tissue^41^ where they are involved in regulating the innate and adaptive immune response. Eosinophils secreted chemokines participate in immunomodulation and tissue remodeling. The correlation between eosinophilia and outcome has been controversial. Some studies reported a positive correlation with survival outcome in colorectal, breast, and prostate cancers^42^ as well as in head and neck cancers, and bladder cancer^43,44^. In other settings, the positive correlation with outcome was confined to early-stage disease, while a negative correlation was noted in advanced-stage diseases like oral squamous cell cancer^45^. Interestingly, tissue infiltrating eosinophils produce CCL11, IL6, and a large amount of TGF-β^46,47^. We hypothesize that our findings of the correlation of eosinophils with poor ICI-based progression-free survival and overall survival in our cohort are related to their production of TGF-β leading to immune suppression. Interaction of tumor-associated macrophages and eosinophils has been postulated^48^. Furthermore, eosinophils have been shown to produce other growth factors such as VEGF. Eosinophils can therefore be implicated in the tumor-related angiogenesis and neovascularization, enhancing the immunosuppressive myeloid phenotype of M2 macrophages and myeloid-derived suppressive cells while also contributing to the formation of connective tissue and desmoplasia around cancer^49^.

**Fig. 5:**
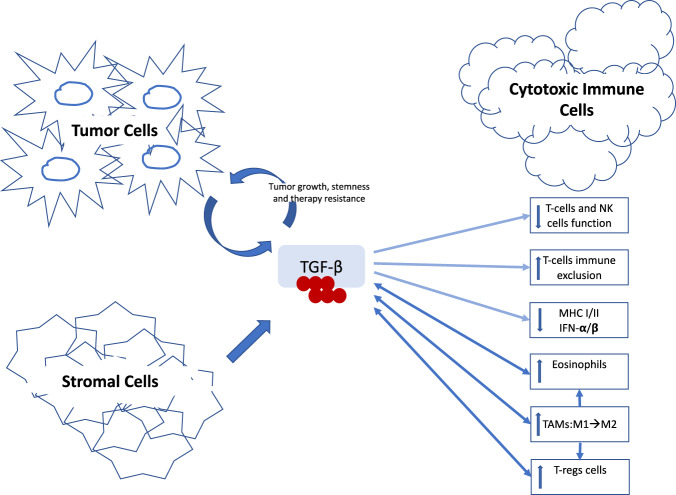
Role of TGF-β in inducing immunosuppressive tumor immune microenvironment. Cartoon diagram of our potential hypothesis showing the impact of TGF-β on promoting immunosuppressive tumor immune microenvironment and potential interaction with other immune cells including tumor-associated macrophages, eosinophils, and T-regulatory cells.

In summary (Fig. 5), we hypothesize that TGF-β is produced by the tumor, T-regulatory cells, and stromal cells resulting in immune suppression of T cells and NK cells, as well as inducing phenotype switch from M1 to M2 macrophages which further amplifying immune suppression by producing TGF-β and recruiting other cells like T-regulatory cells and eosinophils. Eosinophils have been shown to produce large amounts of TGF-β, which further play a role in recruiting and interacting with macrophages to modulate the immunosuppressive microenvironment.

Our study is limited by its retrospective nature, possible heterogenous patient population and small sample size as well as possible selection and confounding biases. The possible variability in therapy, surveillance patterns and follow up were noted and warrant future external validation. As one of the few studies that investigated the transcriptomic profile of the tumor immune microenvironment in gynecologic cancer patients treated with immunotherapy, our study reported interesting findings of the role of immunosuppressive factors may mediate resistance to immunotherapy. The signature can be potentially validated in prospective clinical trials investigating the role of ICI in gynecologic cancer. and potentially used both as a biomarker to predict immunotherapy response and to provide the rationale for combination immunotherapy to target TGF-β to enhance response to immunotherapy in the clinic in a biomarker-based approach. Given recent advances in clinical development of TGF- β inhibitors in the clinic, one additional potential approach is to utilize it in clinical trials assessing those receiving combination of TGF- β and immune checkpoint inhibition.

## Methods

### Patients population and tumor samples

Our patient cohort included endometrial, cervical, and ovarian cancers. The study was approved by the Institutional Review Board and was an exempt from patients’ consent given its retrospective nature. Patient characteristics and available clinical and treatment data of our patients are summarized in Table 1. Median follow up was 12.5 months (2–44 months).

Patients were classified as responders if they had evidence of decreased radiologic tumor burden with a partial or complete response by RECIST 1.1 when evaluated by CT scan per judgment of their treating physician. Patients had to have at least 2 cycles of immunotherapy with interval imaging to assess response compared to pre-treatment imaging. The details and frequency of immune-related toxicities were collected retrospectively from the patient’s medical records. The progression-free survival was calculated from the time of initiation of immunotherapy with immune checkpoint inhibition to disease progression, last follow up or death. Overall survival was calculated from the time of initiation of immunotherapy with immune checkpoint inhibition to death or last follow up. This was done to elucidate the impact of immunotherapy on these survival endpoints.

Formalin-fixed paraffin-embedded (FFPE) tumor samples were collected from the 49 patients at the time of initial diagnosis from primary sites that had been enrolled into this retrospective study with Cleveland Clinic institutional review board approval. The archival FFPE samples were collected when sufficient (>20% tumor content) tumor material was available.

### Next-generation RNA sequencing and bioinformatics pipeline

FFPE specimens were processed and sequenced by MedGenome (Foster City, CA). The archival FFPE samples were used for sequencing when sufficient (>20% tumor content) tumor material was available. All 49 specimens passed RNA extraction QC and RNA library prep QC and proceeded for mRNA sequencing. Based on the quality report of fastq files we trimmed sequence reads wherever necessary to only retain high-quality sequence for further analysis. In addition, the low-quality sequence reads were excluded from the analysis. Data quality checks were performed using FastQC (v0.11.8). The paired-end reads were aligned to the reference human genome Feb. 2009 release downloaded from the UCSC database (GRCh37/hg19). The chromosome fasta file was downloaded from the following website (http://hgdownload.soe.ucsc.edu/goldenPath/hg19/bigZips/chromFa.tar.gz). GTF file was downloaded from the following website (ftp://ftp.ensembl.org/pub/release75/gtf/homo_sapiens/Homo_sapiens.GRCh37.75.gtf.gz). Alignment was performed using STAR (v2.7.3a)^50^. The aligned reads were used for estimating the expression of the genes using HTSeq (v0.11.2). Only reads mapping unambiguously to a single gene were counted, whereas reads aligned to multiple positions or overlapping with more than one gene were discarded. Read count data were normalized and gene expression analysis was performed using R/Bioconductor packages DESeq2 (v1.28.1)^51^. Pathway analysis, gene signature, and upstream regulators were identified using QIAGEN Ingenuity Pathway Analysis (IPA, QIAGEN, Redwood City, CA).

### Gene signature score and immune cell abundance estimation

The signature-based scoring was calculated using the rank-based single-sample gene set scoring method (simpleScore) provided by R package^13^. Singscore method implements a simple single-sample gene-set (gene-signature) scoring method which scores individual samples independently without relying on other samples in gene expression datasets.

Immune cell abundance estimation was based on LM22^52^, which is a signature matrix file consisting of 547 genes that accurately distinguish 22 mature human hematopoietic populations isolated from peripheral blood or in vitro culture conditions, including seven T-cell types, naïve and memory B cells, plasma cells, NK cells, and myeloid subsets. Expression levels for each LM22 cell type were estimated using simpleScore method as described above.

### TGF-β signature validation

We evaluated our 6-gene TGF-β signature for its predictive power of response outcome and overall survival using online tool TIDE: Tumor Immune Dysfunction and Exclusion, which applies custom biomarker gene set to gene expression profiles of 23 cancer studies with immunotherapy, and compared results to existing published biomarkers^53^ (http://tide.dfci.harvard.edu).

### Statistical analysis

We used the two-sided Student’s *t* test test for all comparisons of continuous data and the Spearman correlation coefficient to analyze the correlation between different variables. Fisher’s exact test was used to compare the tumor grade, MSI status, signature score level between different groups. Kaplan–Meier estimation and log-rank tests were used for time-to-event analyses comparing between 2 groups based on individual variables such as response and gene/score with the cohort median value used as a cut-off. Survival analysis on continuous variables such as gene expression was performed using a multivariable Cox proportional hazards model to derive coefficients and *P* values as determined by the default “efron” test. The statistical significance for both pathway analysis and upstream regulator analysis was assessed via Fisher’s exact test by IPA.

All statistical tests were two-sided, and a *p* value of less than 0.05 was considered significant across all analyses performed. Statistical analyses were performed using R (version 4.0.3) and RStudio (version 1.3.1093).

### Reporting summary

Further information on research design is available in the Nature Research Reporting Summary linked to this article.

## Supplementary information

Reporting Summary

Supplementary Information

## Data Availability

The RNA sequencing data used in the current study are deposited in SRA repository (SRP341153) with the following accession number: PRJNA770873.

## Code availability

The codes and software packages are described in the methods section. Any further codes or other data are available upon request.
