# Supplementary material for: High TGF-β signature predicts immunotherapy resistance in gynecologic cancer patients treated with immune checkpoint inhibition
Source: NPJ Precis Oncol. 2021 Dec 17;5:101. doi: 10.1038/s41698-021-00242-8 (PMC8683510; doi:10.1038/s41698-021-00242-8)
Supplement: Supplementary file 2 — Supplementary Information [file 41698_2021_242_MOESM2_ESM.pdf]

Supplementary Table1A: Differentially expressed genes (DEGs) in ovarian cancer patients; B: DEGs in cervical cancer patients.

Gene.names	baseMean	log2 FoldChange	lfcSE	pvalue	padj	Symbol
ENSG00000004776	428.6985	-2.3454	0.6156	0.0001	0.0168	HSPB6
ENSG00000005108	635.1092	2.8147	0.6064	0.0000	0.0016	THSD7A
ENSG00000005189	90.5826	1.6918	0.4176	0.0001	0.0092	REXO5
ENSG00000006459	1122.2799	-1.0243	0.3024	0.0007	0.0442	KDM7A
ENSG00000008735	92.1785	3.8965	0.6882	0.0000	0.0000	MAPK8IP2
ENSG00000011478	60.3820	1.7035	0.4854	0.0004	0.0336	QPCTL
ENSG00000012660	896.6921	-1.3804	0.4112	0.0008	0.0456	ELOVL5
ENSG00000017427	1744.5426	-2.3430	0.6360	0.0002	0.0237	IGF1
ENSG00000029534	279.8104	5.0851	0.7685	0.0000	0.0000	ANK1
ENSG00000042781	168.6755	3.6326	1.0879	0.0008	0.0467	USH2A
ENSG00000056487	113.6435	3.2447	0.7001	0.0000	0.0016	PHF21B
ENSG00000057294	546.7679	-1.2684	0.3546	0.0003	0.0292	PKP2
ENSG00000061455	181.5242	1.8275	0.5325	0.0006	0.0400	PRDM6
ENSG00000064726	701.9953	-1.1707	0.3256	0.0003	0.0285	BTBD1
ENSG00000064787	257.6019	4.3524	0.9596	0.0000	0.0023	BCAS1
ENSG00000069956	717.5501	-1.0642	0.3157	0.0007	0.0452	MAPK6
ENSG00000072201	129.5940	1.9383	0.5779	0.0008	0.0456	LNX1
ENSG00000073150	42.7992	2.4379	0.6666	0.0003	0.0248	PANX2
ENSG00000073282	81.8184	3.0736	0.8501	0.0003	0.0271	TP63
ENSG00000073670	45.9781	1.9923	0.5936	0.0008	0.0456	ADAM11
ENSG00000075643	258.9305	2.9246	0.7863	0.0002	0.0217	MOCOS
ENSG00000076770	1045.4542	-2.2662	0.6761	0.0008	0.0456	MBNL3
ENSG00000079841	157.2805	4.2607	0.9011	0.0000	0.0011	RIMS1
ENSG00000081985	113.1619	3.8746	0.7872	0.0000	0.0006	IL12RB2
ENSG00000084110	99.9567	3.1563	0.6618	0.0000	0.0010	HAL
ENSG00000084674	93.6317	3.3029	0.9762	0.0007	0.0446	APOB
ENSG00000087495	187.6180	3.8049	0.5529	0.0000	0.0000	PHACTR3
ENSG00000088992	27.4727	2.3950	0.6239	0.0001	0.0159	TESC
ENSG00000090104	1851.1716	-2.9576	0.8552	0.0005	0.0375	RGS1
ENSG00000091128	123.7654	4.8295	0.8877	0.0000	0.0001	LAMB4
ENSG00000091137	46.3220	2.9672	0.8663	0.0006	0.0406	SLC26A4
ENSG00000091262	106.2511	2.1807	0.5720	0.0001	0.0168	ABCC6
ENSG00000091490	781.9610	-1.6388	0.4646	0.0004	0.0321	SEL1L3
ENSG00000091536	129.1020	2.5332	0.7125	0.0004	0.0299	MYO15A
ENSG00000091986	3051.3576	-2.7826	0.7650	0.0003	0.0256	CCDC80

ENSG00000095627	48.8305	3.7028	0.7739	0.0000	0.0010	TDRD1
ENSG00000099999	177.7665	1.7453	0.4570	0.0001	0.0166	RNF215
ENSG00000100003	117.3550	1.8805	0.5084	0.0002	0.0231	SEC14L2
ENSG00000100024	66.9609	3.8064	0.8935	0.0000	0.0053	UPB1
ENSG00000100029	309.5620	1.2489	0.3622	0.0006	0.0386	PES1
ENSG00000100101	133.1841	1.7030	0.4258	0.0001	0.0110	NA
ENSG00000100142	209.3371	1.5034	0.4492	0.0008	0.0456	POLR2F
ENSG00000100342	1479.9662	2.9171	0.8168	0.0004	0.0295	APOL1
ENSG00000100346	58.2107	3.0328	0.8337	0.0003	0.0256	CACNA1I
ENSG00000100528	412.8518	-1.2508	0.3406	0.0002	0.0240	CNIH1
ENSG00000101347	2041.0721	-2.0929	0.5365	0.0001	0.0142	SAMHD1
ENSG00000101670	133.9134	2.5482	0.6540	0.0001	0.0142	LIPG
ENSG00000101825	3415.2856	-3.6220	0.5608	0.0000	0.0000	MXRA5
ENSG00000101966	1219.1435	-1.0777	0.3032	0.0004	0.0299	XIAP
ENSG00000102100	204.7741	1.2550	0.3711	0.0007	0.0447	SLC35A2
ENSG00000102466	43.8298	3.5604	0.9432	0.0002	0.0181	FGF14
ENSG00000102760	203.3583	-2.5033	0.7377	0.0007	0.0439	RGCC
ENSG00000104691	295.3141	2.7800	0.7974	0.0005	0.0352	UBXN8
ENSG00000106125	106.9206	1.1306	0.3313	0.0006	0.0421	MINDY4
ENSG00000106278	102.0868	2.7012	0.7521	0.0003	0.0285	PTPRZ1
ENSG00000107317	398.4321	-2.7474	0.7355	0.0002	0.0206	PTGDS
ENSG00000107742	511.8963	-2.1543	0.6435	0.0008	0.0456	SPOCK2
ENSG00000108309	33.0129	2.8973	0.6956	0.0000	0.0066	RUNDC3A
ENSG00000108924	242.2393	2.3123	0.6443	0.0003	0.0285	HLF
ENSG00000109472	520.8158	-2.0515	0.4964	0.0000	0.0072	CPE
ENSG00000110092	1405.1347	-2.0431	0.4886	0.0000	0.0063	CCND1
ENSG00000110395	985.3292	-1.1421	0.3380	0.0007	0.0448	CBL
ENSG00000110921	116.3779	1.4924	0.4094	0.0003	0.0255	MVK
ENSG00000111181	352.2652	2.6964	0.7564	0.0004	0.0299	SLC6A12
ENSG00000111269	743.6443	-1.3501	0.3619	0.0002	0.0209	CREBL2
ENSG00000111843	427.4103	-1.4881	0.4205	0.0004	0.0313	TMEM14C
ENSG00000113361	2607.7795	-3.5552	0.8610	0.0000	0.0072	CDH6
ENSG00000114654	84.3094	2.3109	0.6951	0.0009	0.0487	EFCC1
ENSG00000114850	1703.2331	-1.4046	0.3698	0.0001	0.0171	SSR3
ENSG00000114859	145.7595	1.7208	0.3300	0.0000	0.0002	CLCN2
ENSG00000114948	162.5296	1.9900	0.4485	0.0000	0.0031	ADAM23
ENSG00000115112	2550.6493	5.0536	0.8699	0.0000	0.0000	TFCP2L1
ENSG00000115556	62.1436	2.2522	0.5453	0.0000	0.0072	PLCD4
ENSG00000115561	666.3369	-1.0115	0.2605	0.0001	0.0145	CHMP3
ENSG00000116260	3275.7508	3.1708	0.8138	0.0001	0.0142	QSOX1

ENSG00000116396	472.8946	3.1386	0.7863	0.0001	0.0112	KCNC4
ENSG00000116574	661.0976	-2.2223	0.3360	0.0000	0.0000	RHOU
ENSG00000116885	99.2120	1.3525	0.3483	0.0001	0.0145	OSCP1
ENSG00000117245	36.5791	2.4645	0.5720	0.0000	0.0046	KIF17
ENSG00000117472	701.7623	4.1534	0.9339	0.0000	0.0031	TSPAN1
ENSG00000118156	50.4386	2.9931	0.7781	0.0001	0.0159	ZNF541
ENSG00000119121	87.5359	3.5382	0.8242	0.0000	0.0048	TRPM6
ENSG00000119514	143.9942	-2.9778	0.6770	0.0000	0.0035	GALNT12
ENSG00000119729	1091.5722	-1.5860	0.4714	0.0008	0.0456	RHOQ
ENSG00000123095	1428.7695	-3.0678	0.8360	0.0002	0.0240	BHLHE41
ENSG00000124134	58.4863	3.6063	0.9415	0.0001	0.0161	KCNS1
ENSG00000124783	2778.6538	-1.3553	0.3812	0.0004	0.0299	SSR1
ENSG00000124795	2237.6542	-2.2794	0.6194	0.0002	0.0237	DEK
ENSG00000125249	794.4862	-1.5897	0.4793	0.0009	0.0492	RAP2A
ENSG00000125266	741.0208	-3.0260	0.8510	0.0004	0.0299	EFNB2
ENSG00000125895	40.3182	2.6237	0.6355	0.0000	0.0072	TMEM74B
ENSG00000128242	40.3108	2.6172	0.7786	0.0008	0.0456	GAL3ST1
ENSG00000128683	64.0633	2.8977	0.8287	0.0005	0.0346	GAD1
ENSG00000128989	1984.3449	-1.3945	0.4096	0.0007	0.0429	ARPP19
ENSG00000129128	1392.7238	-1.5163	0.4129	0.0002	0.0240	SPCS3
ENSG00000129696	70.9352	1.5803	0.4770	0.0009	0.0496	TTI2
ENSG00000130307	74.9980	2.5152	0.7249	0.0005	0.0363	USHBP1
ENSG00000130600	7498.8679	-3.6265	0.8585	0.0000	0.0059	H19
ENSG00000130783	47.2979	3.0339	0.8223	0.0002	0.0237	CCDC62
ENSG00000131203	273.5478	4.0147	1.0446	0.0001	0.0159	IDO1
ENSG00000131379	33.2064	2.7497	0.6597	0.0000	0.0066	C3orf20
ENSG00000131400	98.6690	3.5186	0.7831	0.0000	0.0026	NAPSA
ENSG00000131480	61.3103	1.6169	0.4500	0.0003	0.0285	AOC2
ENSG00000131711	1821.5222	-1.7904	0.5213	0.0006	0.0398	MAP1B
ENSG00000132915	79.9619	3.6998	0.9297	0.0001	0.0115	PDE6A
ENSG00000133107	54.0384	2.8439	0.8561	0.0009	0.0489	TRPC4
ENSG00000133110	1568.8073	-3.3290	0.9866	0.0007	0.0449	POSTN
ENSG00000133477	298.8469	2.9931	0.8197	0.0003	0.0252	FAM83F
ENSG00000133488	53.9447	3.2247	0.4034	0.0000	0.0000	SEC14L4
ENSG00000134243	1428.4506	-1.8866	0.5187	0.0003	0.0256	SORT1
ENSG00000134265	609.0682	-1.4023	0.3891	0.0003	0.0280	NAPG
ENSG00000134343	69.1535	3.2263	0.8700	0.0002	0.0224	ANO3
ENSG00000134376	98.7195	3.9236	0.7928	0.0000	0.0006	CRB1
ENSG00000134531	1723.7618	-2.6933	0.6317	0.0000	0.0053	EMP1
ENSG00000134755	2061.4988	-3.5673	0.9218	0.0001	0.0148	DSC2

ENSG00000134851	1353.8550	-1.0364	0.2667	0.0001	0.0145	TMEM165
ENSG00000134970	919.9930	-1.4757	0.4022	0.0002	0.0240	TMED7
ENSG00000135046	2260.7840	-2.1687	0.6107	0.0004	0.0301	ANXA1
ENSG00000135622	157.4893	1.2796	0.3483	0.0002	0.0240	SEMA4F
ENSG00000135740	71.4952	2.0614	0.5055	0.0000	0.0087	SLC9A5
ENSG00000135932	1109.8011	-1.2574	0.3738	0.0008	0.0456	CAB39
ENSG00000136449	94.4480	2.0093	0.5629	0.0004	0.0295	MYCBPAP
ENSG00000136531	189.3697	3.3007	0.8295	0.0001	0.0115	SCN2A
ENSG00000137573	2456.0084	-2.5177	0.6493	0.0001	0.0148	SULF1
ENSG00000137731	356.2081	3.4619	0.8760	0.0001	0.0126	FXYD2
ENSG00000138061	2557.6404	-3.1198	0.8859	0.0004	0.0325	CYP1B1
ENSG00000138386	1204.7047	-1.3636	0.3940	0.0005	0.0372	NAB1
ENSG00000138756	1361.4744	-1.7153	0.4825	0.0004	0.0299	BMP2K
ENSG00000138798	60.7806	3.9816	0.9099	0.0000	0.0038	EGF
ENSG00000139209	81.5025	2.8212	0.8524	0.0009	0.0499	SLC38A4
ENSG00000141337	205.4582	1.1902	0.3474	0.0006	0.0406	ARSG
ENSG00000142606	31.5777	3.1432	0.7745	0.0000	0.0092	MMEL1
ENSG00000143162	1071.1235	-1.6749	0.4057	0.0000	0.0072	CREG1
ENSG00000143320	249.9818	-3.9965	0.6979	0.0000	0.0000	CRABP2
ENSG00000143882	229.9745	2.9919	0.8357	0.0003	0.0290	ATP6V1C2
ENSG00000143995	2903.3624	-2.6905	0.7556	0.0004	0.0299	MEIS1
ENSG00000144810	1731.4979	-2.4501	0.7210	0.0007	0.0434	COL8A1
ENSG00000144837	75.0460	2.5613	0.6816	0.0002	0.0190	PLA1A
ENSG00000145423	1604.6628	-6.2485	1.5434	0.0001	0.0092	SFRP2
ENSG00000147010	726.5962	-1.9051	0.5442	0.0005	0.0344	SH3KBP1
ENSG00000147485	104.4743	3.7387	0.7913	0.0000	0.0011	PXDNL
ENSG00000149527	281.3982	3.9023	0.9163	0.0000	0.0053	PLCH2
ENSG00000150394	66.9885	3.8287	0.7887	0.0000	0.0007	CDH8
ENSG00000150551	393.7716	-3.6231	1.0576	0.0006	0.0406	LYPD1
ENSG00000152380	102.9839	1.6948	0.5055	0.0008	0.0456	FAM151B
ENSG00000152910	38.2718	3.8681	0.8887	0.0000	0.0041	CNTNAP4
ENSG00000152953	114.9373	2.5172	0.7454	0.0007	0.0448	STK32B
ENSG00000153292	207.2775	2.7450	0.7654	0.0003	0.0285	ADGRF1
ENSG00000154269	63.7394	2.8462	0.8491	0.0008	0.0456	ENPP3
ENSG00000154639	713.4484	-2.8068	0.8164	0.0006	0.0394	CXADR
ENSG00000157087	630.6049	3.8104	0.9504	0.0001	0.0107	ATP2B2
ENSG00000157152	55.9608	2.8075	0.8369	0.0008	0.0456	SYN2
ENSG00000157193	205.7421	1.0907	0.3255	0.0008	0.0456	LRP8
ENSG00000158089	221.7139	3.3751	0.9655	0.0005	0.0347	GALNT14
ENSG00000158122	142.9688	-1.6606	0.4900	0.0007	0.0441	PRXL2C

ENSG00000158856	227.6278	2.5403	0.7132	0.0004	0.0299	DMTN
ENSG00000159450	38.4284	2.9256	0.7144	0.0000	0.0081	TCHH
ENSG00000160111	394.5579	1.9049	0.5387	0.0004	0.0315	CPAMD8
ENSG00000160131	846.4933	-1.4153	0.4024	0.0004	0.0329	VMA21
ENSG00000160602	108.1158	1.8000	0.5438	0.0009	0.0499	NEK8
ENSG00000162704	978.3167	-1.4760	0.3415	0.0000	0.0045	ARPC5
ENSG00000162734	1261.3781	-1.7837	0.4966	0.0003	0.0285	PEA15
ENSG00000162779	53.1678	2.9446	0.8720	0.0007	0.0448	AXDND1
ENSG00000162949	408.6067	3.2470	0.8010	0.0001	0.0092	CAPN13
ENSG00000164061	183.0883	2.0224	0.4778	0.0000	0.0057	BSN
ENSG00000164161	113.4269	3.2462	0.8928	0.0003	0.0256	HHIP
ENSG00000164845	25.3760	1.7191	0.4944	0.0005	0.0360	NA
ENSG00000165046	249.0123	2.7695	0.8346	0.0009	0.0490	LETM2
ENSG00000165476	774.7658	-1.5095	0.3995	0.0002	0.0179	REEP3
ENSG00000165810	478.2525	-2.6077	0.5208	0.0000	0.0006	BTNL9
ENSG00000165813	1362.6392	-2.0639	0.6152	0.0008	0.0456	CCDC186
ENSG00000166206	66.2638	2.6596	0.7632	0.0005	0.0353	GABRB3
ENSG00000166535	125.9629	3.8067	0.9043	0.0000	0.0062	A2ML1
ENSG00000166734	1792.1535	-1.3517	0.3927	0.0006	0.0392	CASC4
ENSG00000166750	1442.5703	-1.6254	0.4837	0.0008	0.0456	SLFN5
ENSG00000166819	392.2419	-4.0441	1.1981	0.0007	0.0449	PLIN1
ENSG00000166923	564.5816	-4.2927	1.2374	0.0005	0.0363	GREM1
ENSG00000167100	65.3697	2.1186	0.6002	0.0004	0.0320	SAMD14
ENSG00000167244	38599.4267	-7.5235	1.0892	0.0000	0.0000	IGF2
ENSG00000167693	556.3451	-1.9941	0.4520	0.0000	0.0033	NXN
ENSG00000168389	408.3935	3.2692	0.8128	0.0001	0.0102	MFSD2A
ENSG00000168453	66.4306	2.5703	0.5954	0.0000	0.0045	HR
ENSG00000168517	39.4546	2.5801	0.6144	0.0000	0.0063	HEXIM2
ENSG00000169031	448.2467	3.1215	0.9387	0.0009	0.0487	COL4A3
ENSG00000169220	172.5241	2.4647	0.6698	0.0002	0.0237	RGS14
ENSG00000169247	127.1193	3.3470	0.9375	0.0004	0.0295	SH3TC2
ENSG00000169446	590.2083	-1.6492	0.3665	0.0000	0.0026	MMGT1
ENSG00000169507	89.9744	2.7283	0.7820	0.0005	0.0351	SLC38A11
ENSG00000169862	59.6498	3.2395	0.7276	0.0000	0.0031	CTNND2
ENSG00000170385	809.7892	-1.0984	0.2808	0.0001	0.0142	SLC30A1
ENSG00000170412	373.4952	2.2035	0.6283	0.0005	0.0338	GPRC5C
ENSG00000170419	51.2972	3.4002	0.8876	0.0001	0.0161	VSTM2A
ENSG00000170748	52.4478	3.5712	0.7156	0.0000	0.0006	RBMXL2
ENSG00000170927	1429.6601	3.9647	1.1801	0.0008	0.0456	PKHD1
ENSG00000171236	109.5578	3.7872	0.7675	0.0000	0.0006	LRG1

ENSG00000171509	726.8460	6.3401	0.9371	0.0000	0.0000	RXFP1
ENSG00000171790	41.4747	1.9326	0.4968	0.0001	0.0145	SLFNL1
ENSG00000172264	481.1591	2.4123	0.6548	0.0002	0.0237	MACROD2
ENSG00000172667	1229.6294	-1.4542	0.3468	0.0000	0.0063	ZMAT3
ENSG00000172940	26.7331	2.8784	0.6407	0.0000	0.0026	SLC22A13
ENSG00000173442	1104.0010	-1.4883	0.3373	0.0000	0.0033	EHBP1L1
ENSG00000173848	953.4207	-1.5219	0.3486	0.0000	0.0039	NET1
ENSG00000174514	80.0972	2.6406	0.7168	0.0002	0.0237	MFSD4A
ENSG00000175445	628.7944	-3.8033	0.9930	0.0001	0.0161	LPL
ENSG00000175701	231.6076	3.9192	0.9032	0.0000	0.0042	MTLN
ENSG00000175793	111.3804	4.5603	0.8970	0.0000	0.0004	SFN
ENSG00000176641	1026.8416	-2.0812	0.5684	0.0003	0.0245	RNF152
ENSG00000176697	57.7769	2.4316	0.6909	0.0004	0.0327	BDNF
ENSG00000176845	224.8137	-2.1116	0.4332	0.0000	0.0007	METRNL
ENSG00000176920	107.7943	2.6434	0.7721	0.0006	0.0406	FUT2
ENSG00000177359	135.8792	3.3600	0.7409	0.0000	0.0023	OVOS2
ENSG00000177380	82.2276	2.4844	0.6910	0.0003	0.0285	PPFIA3
ENSG00000178460	96.8348	2.8246	0.8437	0.0008	0.0456	MCMDC2
ENSG00000178732	26.0978	2.8208	0.8325	0.0007	0.0441	GP5
ENSG00000179833	696.6794	-1.1932	0.2869	0.0000	0.0067	SERTAD2
ENSG00000180438	48.3173	2.4668	0.7157	0.0006	0.0387	TPRXL
ENSG00000180447	594.0791	-3.0558	0.8047	0.0001	0.0171	GAS1
ENSG00000181039	33.7784	2.6967	0.7841	0.0006	0.0394	NA
ENSG00000181143	33879.3221	-3.2565	0.8285	0.0001	0.0135	MUC16
ENSG00000181704	943.6172	-1.3413	0.4005	0.0008	0.0456	YIPF6
ENSG00000183230	108.8694	2.3465	0.7068	0.0009	0.0489	CTNNA3
ENSG00000183914	204.4704	2.2487	0.5970	0.0002	0.0185	DNAH2
ENSG00000184937	1390.5850	-3.0923	0.9118	0.0007	0.0439	WT1
ENSG00000185008	464.0077	3.2323	0.8248	0.0001	0.0141	ROBO2
ENSG00000185100	136.3376	2.6695	0.6832	0.0001	0.0142	ADSS1
ENSG00000185432	813.0562	-2.1083	0.5778	0.0003	0.0253	METTL7A
ENSG00000185664	82.7484	3.3699	0.8767	0.0001	0.0159	PMEL
ENSG00000186530	18.8209	2.3078	0.6834	0.0007	0.0448	NA
ENSG00000186976	198.7234	1.6479	0.4355	0.0002	0.0179	EFCAB6
ENSG00000187720	1769.8301	-3.3115	0.8171	0.0001	0.0092	THSD4
ENSG00000187775	152.7760	3.7318	0.9109	0.0000	0.0081	DNAH17
ENSG00000187860	75.4399	2.2363	0.4602	0.0000	0.0007	CCDC157
ENSG00000188042	911.4367	-2.9674	0.5993	0.0000	0.0006	ARL4C
ENSG00000188825	529.9912	1.8469	0.3896	0.0000	0.0011	LINC00910
ENSG00000188897	158.9894	2.5215	0.6394	0.0001	0.0129	LOC40049

						9
ENSG00000188993	31.6002	2.0236	0.6007	0.0008	0.0452	LRRC66
ENSG00000189350	146.4123	2.6808	0.8007	0.0008	0.0456	TOGARAM2
ENSG00000196277	122.8402	3.4515	0.7081	0.0000	0.0007	GRM7
ENSG00000196456	131.7097	1.4098	0.4030	0.0005	0.0346	ZNF775
ENSG00000196559	31.0899	2.0000	0.5255	0.0001	0.0168	NA
ENSG00000196839	122.2615	2.4752	0.7126	0.0005	0.0362	ADA
ENSG00000196862	24.4476	3.0283	0.7956	0.0001	0.0168	RGPD4
ENSG00000196937	575.5112	-1.2918	0.2874	0.0000	0.0026	FAM3C
ENSG00000197329	591.3309	-1.5161	0.4121	0.0002	0.0237	PELI1
ENSG00000197705	352.0954	-2.7918	0.7401	0.0002	0.0182	KLHL14
ENSG00000197892	1564.0692	2.3630	0.7110	0.0009	0.0488	KIF13B
ENSG00000197932	52.4583	-1.7166	0.4440	0.0001	0.0149	NA
ENSG00000198691	289.4154	4.0175	0.8264	0.0000	0.0007	ABCA4
ENSG00000203734	37.8109	3.1743	0.8841	0.0003	0.0285	ECT2L
ENSG00000203999	59.8673	2.6988	0.7645	0.0004	0.0320	LINC01270
ENSG00000204323	87.6890	2.6610	0.6796	0.0001	0.0141	SMIM5
ENSG00000204370	382.7973	-1.5464	0.4457	0.0005	0.0363	SDHD
ENSG00000205795	516.8695	4.4651	0.9198	0.0000	0.0007	CYS1
ENSG00000207263	39.1892	2.5078	0.7369	0.0007	0.0429	SNORD116-16
ENSG00000211455	1055.8348	-1.7636	0.4664	0.0002	0.0179	STK38L
ENSG00000212402	2241.9046	4.6561	0.9881	0.0000	0.0012	SNORA74B
ENSG00000213071	25.1888	2.7254	0.7338	0.0002	0.0220	LPAL2
ENSG00000213411	26.1017	1.8274	0.5455	0.0008	0.0456	NA
ENSG00000214145	30.8826	2.4604	0.6763	0.0003	0.0256	LINC00887
ENSG00000214562	28.4121	2.0019	0.4033	0.0000	0.0006	NUTM2D
ENSG00000214711	84.9796	3.9369	0.9145	0.0000	0.0046	CAPN14
ENSG00000215114	744.9809	-1.2928	0.3804	0.0007	0.0434	UBXN2B
ENSG00000224577	20.3229	2.3169	0.6081	0.0001	0.0168	LINC01117
ENSG00000224812	23.6925	2.2094	0.6677	0.0009	0.0499	TMEM72-AS1
ENSG00000226067	27.7093	1.8373	0.5088	0.0003	0.0275	NA
ENSG00000226328	126.2462	2.2738	0.6286	0.0003	0.0270	NUP50-DT
ENSG00000226763	21.0746	2.3138	0.5806	0.0001	0.0115	SRRM5
ENSG00000227242	33.0018	2.4212	0.7184	0.0008	0.0452	NA
ENSG00000227248	90.8520	2.3611	0.6043	0.0001	0.0142	NA
ENSG00000228113	33.7441	3.0775	0.8455	0.0003	0.0256	NA
ENSG00000228463	39.0178	3.0899	0.5243	0.0000	0.0000	NA
ENSG00000228477	36.3815	3.1259	0.8118	0.0001	0.0158	NA

ENSG00000229021	75.0353	4.7206	0.9834	0.0000	0.0009	NA
ENSG00000229233	22.9908	2.7751	0.7732	0.0003	0.0285	NA
ENSG00000229980	57.0899	2.5767	0.7759	0.0009	0.0489	TOB1-AS1
ENSG00000230510	68.2751	2.1544	0.6172	0.0005	0.0350	PPP5D1
ENSG00000230922	80.3449	3.4416	0.8125	0.0000	0.0057	LOC10192 7551
ENSG00000231407	32.7311	2.9547	0.8672	0.0007	0.0428	NA
ENSG00000231419	58.9174	3.1952	0.8455	0.0002	0.0179	LINC00689
ENSG00000232759	48.1268	3.5170	0.7741	0.0000	0.0023	LOC10050 6178
ENSG00000233786	30.0756	3.8593	0.9490	0.0000	0.0090	NA
ENSG00000233791	33.2053	2.8587	0.8274	0.0006	0.0378	LINC01136
ENSG00000233885	19.5267	2.4233	0.6974	0.0005	0.0362	NA
ENSG00000234685	690.5897	3.6935	1.0192	0.0003	0.0266	NA
ENSG00000235655	428.1020	-2.3769	0.6086	0.0001	0.0142	NA
ENSG00000235920	36.5045	2.8962	0.6324	0.0000	0.0020	NA
ENSG00000236753	50.1236	2.5493	0.5963	0.0000	0.0051	MKLN1-AS
ENSG00000236824	128.8669	3.3965	0.9764	0.0005	0.0360	BCYRN1
ENSG00000236830	135.6245	1.9995	0.5671	0.0004	0.0322	CBR3-AS1
ENSG00000237517	93.6268	3.9387	0.7389	0.0000	0.0001	NA
ENSG00000239039	671.9930	3.3173	0.9251	0.0003	0.0285	SNORD13
ENSG00000239282	32.2821	2.1188	0.5436	0.0001	0.0142	CASTOR1
ENSG00000239455	19.8531	2.8758	0.7431	0.0001	0.0148	NA
ENSG00000244479	92.7709	1.4378	0.4047	0.0004	0.0301	NA
ENSG00000244607	45.6092	2.2603	0.6771	0.0008	0.0467	CCDC13
ENSG00000246089	29.7926	1.8230	0.5033	0.0003	0.0267	LOC10028 7015
ENSG00000249700	56.9147	3.6771	0.8782	0.0000	0.0063	SRD5A3-AS1
ENSG00000249859	643.6567	1.7429	0.4500	0.0001	0.0148	PVT1
ENSG00000253525	27.1026	2.8792	0.7497	0.0001	0.0159	NA
ENSG00000253669	65.7799	3.6836	0.9637	0.0001	0.0165	NA
ENSG00000256222	23.1337	2.7579	0.7901	0.0005	0.0350	MTRNR2L 3
ENSG00000257108	17.6044	2.3125	0.5214	0.0000	0.0031	NHLRC4
ENSG00000261115	154.9425	3.5738	0.8501	0.0000	0.0062	TMEM178 B
ENSG00000262468	53.5640	2.4392	0.6604	0.0002	0.0234	NA
ENSG00000263006	997.1795	4.0846	1.0305	0.0001	0.0122	ROCK1P1
ENSG00000266208	39.4023	3.3122	0.7911	0.0000	0.0063	NA
ENSG00000266821	20.7038	2.5500	0.7570	0.0008	0.0452	NA
ENSG00000267586	54.5697	3.0233	0.8875	0.0007	0.0428	LINC00907
ENSG00000268416	95.1843	3.1095	0.7435	0.0000	0.0063	NA

ENSG00000268423	15.6673	2.3652	0.6639	0.0004	0.0299	NA
ENSG00000272476	26.8593	3.1863	0.9392	0.0007	0.0439	NA
ENSG00000272761	1338.4303	-3.2436	0.8523	0.0001	0.0168	NA

Supplementary Table1B: Differentially expressed genes (DEGs) in cervical cancer patients.

Gene.names	baseMean	log2 FoldChange	lfcSE	pvalue	padj	Symbol
ENSG00000006074	386.7589	-4.5684	1.2280	0.0002	0.0333	CCL18
ENSG00000007306	85.9209	-5.1083	1.1181	0.0000	0.0026	CEACAM7
ENSG00000008735	136.3932	-3.0820	0.6168	0.0000	0.0004	MAPK8IP2
ENSG00000011201	213.0367	-2.3866	0.5797	0.0000	0.0115	KAL1
ENSG00000019186	1309.3708	-6.3259	1.2853	0.0000	0.0006	CYP24A1
ENSG00000022267	1559.4032	3.6638	0.8398	0.0000	0.0052	FHL1
ENSG00000037965	77.7197	-4.5548	0.8885	0.0000	0.0003	HOXC8
ENSG00000053108	82.3246	-3.9698	1.0655	0.0002	0.0329	FSTL4
ENSG00000054690	444.0317	-2.4132	0.6697	0.0003	0.0474	PLEKHH1
ENSG00000058085	3084.2877	-4.7389	1.0913	0.0000	0.0055	LAMC2
ENSG00000060718	566.3015	-4.2850	1.1169	0.0001	0.0226	COL11A1
ENSG00000064270	409.5769	-5.8666	1.2668	0.0000	0.0021	ATP2C2
ENSG00000065618	1903.1288	-6.7888	1.2465	0.0000	0.0001	COL17A1
ENSG00000073150	144.7197	-4.9416	1.2676	0.0001	0.0191	PANX2
ENSG00000074276	134.3672	-6.0629	1.6552	0.0002	0.0397	CDHR2
ENSG00000077157	6189.3140	2.6452	0.7178	0.0002	0.0378	PPP1R12B
ENSG00000078081	521.8019	-3.3605	0.8623	0.0001	0.0191	LAMP3
ENSG00000080854	312.7429	3.0977	0.7979	0.0001	0.0201	IGSF9B
ENSG00000091831	2266.7158	2.9309	0.8147	0.0003	0.0476	ESR1
ENSG00000095203	245.6703	-2.8517	0.7399	0.0001	0.0213	EPB41L4B
ENSG00000099953	816.9874	-3.4568	0.8806	0.0001	0.0177	MMP11
ENSG00000100065	477.1702	-4.2889	0.7527	0.0000	0.0000	CARD10
ENSG00000100344	105.6326	-4.2087	0.7623	0.0000	0.0001	PNPLA3
ENSG00000101144	609.3605	-4.8587	1.0941	0.0000	0.0044	BMP7
ENSG00000101187	365.8460	-2.9718	0.7550	0.0001	0.0177	SLCO4A1
ENSG00000102934	52.4235	-3.8770	0.7408	0.0000	0.0002	PLLP
ENSG00000103888	550.6535	-3.1015	0.8568	0.0003	0.0449	KIAA1199
ENSG00000108244	322.2949	-3.8383	0.5500	0.0000	0.0000	KRT23
ENSG00000108375	509.7523	-2.5993	0.7093	0.0002	0.0397	RNF43
ENSG00000113790	334.9415	-2.0981	0.5715	0.0002	0.0396	EHHADH
ENSG00000115221	1569.1785	-4.1626	0.7853	0.0000	0.0002	ITGB6

ENSG00000115616	148.7139	-4.8452	1.1565	0.0000	0.0093	SLC9A2
ENSG00000116774	383.9937	2.9416	0.7777	0.0002	0.0276	OLFML3
ENSG00000119547	285.4574	-2.9480	0.7285	0.0001	0.0138	ONECUT2
ENSG00000119917	611.8216	-2.3751	0.5567	0.0000	0.0071	IFIT3
ENSG00000121413	393.8476	2.2376	0.6143	0.0003	0.0420	ZSCAN18
ENSG00000123358	2785.4060	4.1980	0.5528	0.0000	0.0000	NR4A1
ENSG00000123500	168.2643	-3.1927	0.6284	0.0000	0.0003	COL10A1
ENSG00000124102	1077.0534	-5.5348	1.4055	0.0001	0.0177	PI3
ENSG00000125744	95.1477	-3.1715	0.7641	0.0000	0.0103	RTN2
ENSG00000126778	121.0625	-4.3957	1.1363	0.0001	0.0207	SIX1
ENSG00000127533	52.9149	-3.3969	0.8118	0.0000	0.0094	F2RL3
ENSG00000129451	624.7723	-4.7132	1.1275	0.0000	0.0094	KLK10
ENSG00000130176	1075.0776	4.4424	0.8535	0.0000	0.0002	CNN1
ENSG00000132000	107.0373	-2.7267	0.5614	0.0000	0.0008	PODNL1
ENSG00000132329	134.3240	3.3337	0.7833	0.0000	0.0072	RAMP1
ENSG00000132517	143.0001	-5.0152	1.1012	0.0000	0.0027	SLC52A1
ENSG00000133477	2029.9352	-6.4971	1.0536	0.0000	0.0000	FAM83F
ENSG00000135722	179.7716	-2.1078	0.5439	0.0001	0.0204	FBXL8
ENSG00000137745	446.7221	-6.3086	1.6870	0.0002	0.0318	MMP13
ENSG00000142661	154.3812	-4.5243	1.1322	0.0001	0.0157	MYOM3
ENSG00000142871	1934.0197	3.3502	0.8222	0.0000	0.0127	CYR61
ENSG00000143196	403.6872	4.0238	0.9830	0.0000	0.0121	DPT
ENSG00000148180	4604.7548	2.2161	0.5176	0.0000	0.0069	GSN
ENSG00000148848	1080.1877	-2.6941	0.6157	0.0000	0.0050	ADAM12
ENSG00000149948	980.3121	-5.4404	1.2428	0.0000	0.0050	HMGA2
ENSG00000150394	158.1227	-4.6296	1.2394	0.0002	0.0320	CDH8
ENSG00000151012	2172.8371	-6.3940	1.2185	0.0000	0.0002	SLC7A11
ENSG00000151632	3265.9393	-6.6863	1.3752	0.0000	0.0008	AKR1C2
ENSG00000151692	986.0388	2.2125	0.5113	0.0000	0.0057	RNF144A
ENSG00000154734	1364.2685	2.5734	0.6526	0.0001	0.0177	ADAMTS1
ENSG00000154764	110.2004	-3.4335	0.7764	0.0000	0.0045	WNT7A
ENSG00000155265	170.5601	-4.4921	1.2284	0.0003	0.0403	GOLGA7B
ENSG00000157551	662.9991	-4.5202	0.8469	0.0000	0.0001	KCNJ15
ENSG00000159374	70.9439	-4.0646	1.0765	0.0002	0.0280	M1AP
ENSG00000162105	528.7574	-3.5541	0.8796	0.0001	0.0138	SHANK2
ENSG00000162772	777.4684	2.5983	0.6563	0.0001	0.0169	ATF3
ENSG00000162849	774.6338	-3.0226	0.5842	0.0000	0.0002	KIF26B
ENSG00000163431	1000.5201	3.4412	0.8587	0.0001	0.0152	LMOD1
ENSG00000164078	704.6886	-3.5448	0.8069	0.0000	0.0049	MST1R
ENSG00000164379	524.3936	-5.7534	1.0304	0.0000	0.0000	FOXQ1

ENSG00000164683	159.9631	-2.7830	0.6977	0.0001	0.0160	HEY1
ENSG00000164929	224.8398	-4.4194	1.0787	0.0000	0.0121	BAALC
ENSG00000165816	79.8524	-4.2002	0.9442	0.0000	0.0043	VWA2
ENSG00000165949	1360.4151	-3.1194	0.8168	0.0001	0.0241	IFI27
ENSG00000166402	457.8974	2.8266	0.7801	0.0003	0.0447	TUB
ENSG00000166415	583.3642	-5.8481	0.9055	0.0000	0.0000	WDR72
ENSG00000167723	436.7205	-3.0364	0.7740	0.0001	0.0177	TRPV3
ENSG00000167757	264.0417	-5.2252	1.0019	0.0000	0.0002	KLK11
ENSG00000168477	762.9540	2.3911	0.6651	0.0003	0.0477	TNXB
ENSG00000170537	267.5489	-3.6541	0.7277	0.0000	0.0004	TMC7
ENSG00000172935	220.6779	3.0027	0.7367	0.0000	0.0127	MRGPRF
ENSG00000175164	136.2854	-3.7355	0.9917	0.0002	0.0288	ABO
ENSG00000176920	271.7605	-5.4923	0.6684	0.0000	0.0000	FUT2
ENSG00000179044	58.3255	-3.3005	0.8018	0.0000	0.0115	EXOC3L1
ENSG00000181333	690.5668	-6.7092	1.5259	0.0000	0.0049	HEPHL1
ENSG00000183091	798.2250	-4.0034	1.0366	0.0001	0.0211	NEB
ENSG00000184731	983.8972	-3.3323	0.7672	0.0000	0.0055	FAM110C
ENSG00000184792	274.6031	-2.6605	0.6770	0.0001	0.0177	OSBP2
ENSG00000185436	169.2463	-2.6137	0.6566	0.0001	0.0163	IFNLR1
ENSG00000185630	5978.0446	2.3274	0.6343	0.0002	0.0396	PBX1
ENSG00000186395	1166.3357	-4.5107	1.1363	0.0001	0.0166	KRT10
ENSG00000197261	209.1753	-2.6989	0.6422	0.0000	0.0090	C6orf141
ENSG00000198796	251.2740	-3.9813	1.0142	0.0001	0.0177	ALPK2
ENSG00000198829	123.8269	-3.7752	0.9795	0.0001	0.0213	SUCNR1
ENSG00000203995	222.8689	-3.9180	0.9676	0.0001	0.0138	ZYG11A
ENSG00000204175	217.1283	-4.8018	0.9410	0.0000	0.0003	GPRIN2
ENSG00000204618	109.1314	-2.4027	0.6673	0.0003	0.0474	RNF39
ENSG00000206195	231.2412	-3.8959	0.8066	0.0000	0.0008	AP000525.9
ENSG00000208035	46.3475	4.2981	1.0459	0.0000	0.0117	MIR143
ENSG00000211650	74.0082	4.3672	0.9873	0.0000	0.0045	IGLV5-45
ENSG00000213088	84.7927	2.3219	0.6376	0.0003	0.0420	DARC
ENSG00000214776	112.6147	-3.9779	0.9861	0.0001	0.0140	RP11-726G1.1
ENSG00000225210	107.2224	-3.5199	0.8231	0.0000	0.0069	AL589743.1
ENSG00000233818	70.0878	-3.4968	0.8422	0.0000	0.0103	AP000695.4
ENSG00000237978	105.5481	-3.4417	0.5517	0.0000	0.0000	RP11-385J1.2
ENSG00000238961	55.2010	2.8561	0.7188	0.0001	0.0165	SNORA47
ENSG00000246334	37.0780	-2.9699	0.7577	0.0001	0.0178	PRR7-AS1

ENSG00000251003	108.4283	-3.1681	0.8848	0.0003	0.0500	
ENSG00000253007	32.4161	2.8300	0.7002	0.0001	0.0138	SNORA76
ENSG00000253864	2059.3371	3.4833	0.8661	0.0001	0.0145	AC131025.8
ENSG00000258947	102.8474	-4.0501	0.6527	0.0000	0.0000	TUBB3
ENSG00000260027	78.1790	-3.0194	0.6549	0.0000	0.0022	HOXB7
ENSG00000260597	66.2963	-3.5156	0.7301	0.0000	0.0009	AC012531.25
ENSG00000260859	70.1007	3.6492	0.7247	0.0000	0.0004	RP11-254F19.2
ENSG00000263142	91.5630	-3.6310	0.9166	0.0001	0.0169	LRRC37A17P
ENSG00000272796	44.7950	-2.8406	0.7238	0.0001	0.0177	RP1-74M1.3

Supplemental Table 2. Canonical pathways curated based on differentially expressed genes using IPA.

**Ingenuity Canonical Pathways for Entire Cohort**	**-log(p-value)**	**p-value**	**Ratio**	**Molecules**
Glioma Signaling	2.91	0.0012	0.0182	CDKN2B,PRKCI
Xenobiotic Metabolism General Signaling Pathway	2.68	0.0021	0.014	NQO1,PRKCI
Role of Pattern Recognition Receptors in Recognition of Bacteria and Viruses	2.62	0.0024	0.013	OAS2,PRKCI
NRF2-mediated Oxidative Stress Response	2.44	0.0036	0.0106	NQO1,PRKCI
RAR Activation	2.42	0.0038	0.0103	CRABP2,PRKCI
Superoxide Radicals Degradation	2.41	0.0039	0.125	NQO1
Systemic Lupus Erythematosus In B Cell Signaling Pathway	2.13	0.0074	0.00727	IFIT3,PRKCI
Xenobiotic Metabolism Signaling	2.09	0.0081	0.00697	NQO1,PRKCI
Molecular Mechanisms of Cancer	1.82	0.0151	0.005	CDKN2B,PRKCI
Circadian Rhythm Signaling	1.8	0.0158	0.0303	BHLHE41
Interferon Signaling	1.76	0.0174	0.0278	IFIT3
Mechanisms of Viral Exit from Host Cells	1.71	0.0195	0.0244	PRKCI
nNOS Signaling in Neurons	1.65	0.0224	0.0213	PRKCI
UVC-Induced MAPK Signaling	1.61	0.0245	0.0196	PRKCI
UVB-Induced MAPK	1.6	0.0251	0.0192	PRKCI

Signaling				
Retinoic acid Mediated Apoptosis Signaling	1.54	0.0288	0.0167	CRABP2
Thrombopoietin Signaling	1.52	0.0302	0.0159	PRKCI
Cell Cycle: G1/S Checkpoint Regulation	1.5	0.0316	0.0149	CDKN2B
ErbB4 Signaling	1.5	0.0316	0.0149	PRKCI
Calcium-induced T Lymphocyte Apoptosis	1.5	0.0316	0.0152	PRKCI
Nur77 Signaling in T Lymphocytes	1.49	0.0324	0.0147	PRKCI
Growth Hormone Signaling	1.47	0.0339	0.0141	PRKCI
Small Cell Lung Cancer Signaling	1.47	0.0339	0.0141	CDKN2B
Melatonin Signaling	1.46	0.0347	0.0139	PRKCI
Hypoxia Signaling in the Cardiovascular System	1.45	0.0355	0.0135	NQO1
Macropinocytosis Signaling	1.44	0.0363	0.0132	PRKCI
NF-κB Activation by Viruses	1.44	0.0363	0.0132	PRKCI
VDR/RXR Activation	1.43	0.0372	0.0128	PRKCI
IL-3 Signaling	1.42	0.0380	0.0127	PRKCI
Cyclins and Cell Cycle Regulation	1.41	0.0389	0.0123	CDKN2B
Prolactin Signaling	1.41	0.0389	0.0123	PRKCI
HER-2 Signaling in Breast Cancer	1.4	0.0398	0.0119	PRKCI
VEGF Family Ligand-Receptor Interactions	1.4	0.0398	0.0119	PRKCI
LPS-stimulated MAPK Signaling	1.4	0.0398	0.012	PRKCI
Xenobiotic Metabolism AHR Signaling Pathway	1.39	0.0407	0.0118	NQO1
CCR5 Signaling in Macrophages	1.35	0.0447	0.0106	PRKCI
ErbB Signaling	1.35	0.0447	0.0106	PRKCI
Fcγ Receptor-mediated Phagocytosis in Macrophages and Monocytes	1.35	0.0447	0.0106	PRKCI
α-Adrenergic Signaling	1.34	0.0457	0.0104	PRKCI
Apelin Cardiomyocyte Signaling Pathway	1.33	0.0468	0.0101	PRKCI
Nitric Oxide Signaling in the Cardiovascular System	1.33	0.0468	0.0101	PRKCI
Virus Entry via Endocytic Pathways	1.32	0.0479	0.0098	PRKCI
Neuropathic Pain Signaling In Dorsal Horn Neurons	1.32	0.0479	0.0099	PRKCI

IGF-1 Signaling	1.31	0.0490	0.00962	PRKCI

**Ingenuity Canonical Pathways for Ovarian Cancer Patients**	**-log(p-value)**	**p-value**	**Ratio**	**Molecules**
Glioblastoma Multiforme Signaling	3.92	0.0001	0.0545	CCND1,EGF,IGF1,IGF2,PLCD4,PLCH2,RAP2A,RHOQ,RHOU
Phospholipases	3.06	0.0009	0.0769	LIPG,LPL,PLA1A,PLCD4,PLCH2
Synaptogenesis Signaling Pathway	3.02	0.0010	0.0353	ARPC5,BDNF,CDH6,CDH8,EFNB2,GRM7,LRP8,MAP1B,NAPG,RAP2A,SYN2
Clathrin-mediated Endocytosis Signaling	2.76	0.0017	0.0415	APOB,APOL1,ARPC5,CBL,EGF,FGF14,IGF1,SH3KBP1
Tumor Microenvironment Pathway	2.38	0.0042	0.0398	CCND1,EGF,FGF14,IDO1,IGF1,IGF2,RAP2A
Axonal Guidance Signaling	2.34	0.0046	0.0263	ADAM11,ADAM23,ARPC5,BDNF,EFNB2,EGF,HHIP,IGF1,PLCD4,PLCH2,RAP2A,ROBO2,SEMA4F
Synaptic Long Term Depression	2.22	0.0060	0.037	CACNA1I,GAD1,GRM7,IGF1,PLCD4,PLCH2,RAP2A
Glioma Signaling	2.07	0.0085	0.0455	CCND1,EGF,IGF1,IGF2,RAP2A
Thio-molybdenum Cofactor Biosynthesis	1.94	0.0115	1	MOCOS
Thyroid Cancer Signaling	1.9	0.0126	0.0506	BDNF,CCND1,IGF1,RAP2A
Actin Nucleation by ARP-WASP Complex	1.86	0.0138	0.0494	ARPC5,RAP2A,RHOQ,RHOU
14-3-3-mediated Signaling	1.82	0.0151	0.0394	CBL,PLCD4,PLCH2,RAP2A,SFN
Endocannabinoid Neuronal Synapse Pathway	1.8	0.0158	0.0391	CACNA1I,MAPK6,PLCD4,PLCH2,RIMS1
Triacylglycerol Degradation	1.74	0.0182	0.0612	LIPG,LPL,PLA1A
Insulin Secretion Signaling Pathway	1.65	0.0224	0.0287	CPE,NAPG,PLCD4,PLCH2,SPCS3,SSR1,SSR3
Glutamate Dependent Acid Resistance	1.64	0.0229	0.5	GAD1
GABA Receptor Signaling	1.63	0.0234	0.0421	CACNA1I,GABRB3,GAD1,SLC6A12
Bladder Cancer Signaling	1.6	0.0251	0.0412	CCND1,EGF,FGF14,RAP2A
p53 Signaling	1.59	0.0257	0.0408	CCND1,SFN,TP63,WT1
Apelin Cardiomyocyte Signaling Pathway	1.57	0.0269	0.0404	MAPK6,PLCD4,PLCH2,SLC9A5
Neuropathic Pain Signaling In Dorsal Horn Neurons	1.55	0.0282	0.0396	BDNF,GRM7,PLCD4,PLCH2
Thrombin Signaling	1.49	0.0324	0.0288	EGF,PLCD4,PLCH2,RAP2A,RHOQ,RHOU
Wnt/Ca+ pathway	1.47	0.0339	0.0484	PDE6A,PLCD4,PLCH2

Thymine Degradation	1.47	0.0339	0.333	UPB1
Uracil Degradation II (Reductive)	1.47	0.0339	0.333	UPB1
Estrogen Receptor Signaling	1.46	0.0347	0.0244	CCND1,EGF,IGF1,IGF2,PLCD4,PLCH2,RAP2A,SDHD
Sphingosine-1-phosphate Signaling	1.35	0.0447	0.0342	PLCD4,PLCH2,RHOQ,RHOU
Phenylethylamine Degradation I	1.35	0.0447	0.25	AOC2
ERK5 Signaling	1.31	0.0490	0.0417	EGF,RAP2A,SFN

**Ingenuity Canonical Pathways for Cervical Cancer Patients**	**-log(p-value)**	**p-value**	**Ratio**	**Molecules**
Inhibition of Matrix Metalloproteases	3.15	0.0007	0.0769	ADAM12,MMP11,MMP13
Intrinsic Prothrombin Activation Pathway	3.05	0.0009	0.0714	COL10A1,KLK10,KLK11
Axonal Guidance Signaling	2.76	0.0017	0.0162	ADAM12,ADAMTS1,BMP7,MMP11,MMP13,SHANK2,TUBB3,WNT7A
GP6 Signaling Pathway	2.69	0.0020	0.0336	COL10A1,COL11A1,COL17A1,LAMC2
MSP-RON Signaling Pathway	2.65	0.0022	0.0517	KLK10,KLK11,MST1R
HOTAIR Regulatory Pathway	2.23	0.0059	0.025	ESR1,MMP11,MMP13,WNT7A
Hepatic Fibrosis / Hepatic Stellate Cell Activation	2	0.0100	0.0215	COL10A1,COL11A1,COL17A1,MMP13
MSP-RON Signaling In Macrophages Pathway	1.84	0.0145	0.0265	KLK10,KLK11,MST1R
Role of Osteoblasts, Osteoclasts and Chondrocytes in Rheumatoid Arthritis	1.77	0.0170	0.0183	BMP7,GSN,MMP13,WNT7A
Fatty Acid β-oxidation III (Unsaturated, Odd Number)	1.75	0.0178	0.25	EHHADH
Atherosclerosis Signaling	1.71	0.0195	0.0236	COL10A1,MMP13,PNPLA3
MSP-RON Signaling In Cancer Cells Pathway	1.65	0.0224	0.0224	KLK10,KLK11,MST1R
SPINK1 Pancreatic Cancer Pathway	1.53	0.0295	0.0333	KLK10,KLK11
Basal Cell Carcinoma Signaling	1.38	0.0417	0.0278	BMP7,WNT7A
Granulocyte Adhesion and Diapedesis	1.37	0.0427	0.0174	CCL18,MMP11,MMP13
Calcium Transport I	1.36	0.0437	0.1	ATP2C2

Supplementary Table 3: Genes curated for TGF-β signaling and their association with patients response to immunotherapy.

GeneSymbol	pvalue
SLC20A1	0.022
XIAP	0.023
TGFBR1	0.047
BMPR2	0.081
FKBP1A	0.09
SKIL	0.09
RHOA	0.1
SERPINE1	0.106
SMURF2	0.118
RAB31	0.124
LEFTY2	0.13
MAP3K7	0.144
CTNNB1	0.151
TJP1	0.151
BMP2	0.167
IFNGR2	0.201
TRIM33	0.201
UBE2D3	0.201
KLF10	0.211
ARID4B	0.22
CREBBP	0.241
EP300	0.241
LTBP2	0.251
CDH1	0.262
ID1	0.262
PPP1CA	0.297
PPM1A	0.322
SKI	0.322
FURIN	0.335
HIPK2	0.348
FNTA	0.375
ID2	0.389
APC	0.419
BCAR3	0.419
MAP2K1	0.419

SMAD2	0.434
TGIF1	0.481
NCOR2	0.498
THBS1	0.498
MAPK3	0.514
ID3	0.531
SMAD7	0.531
WWTR1	0.531
TGFBR2	0.549
PPP1R15A	0.566
SMAD6	0.566
SMURF1	0.584
ENG	0.62
ZFYVE9	0.62
TAB1	0.715
ACVR1	0.735
CDKN1C	0.754
SPTBN1	0.754
SMAD1	0.794
SMAD3	0.814
NOG	0.835
BMPR1A	0.855
TGFB2	0.876
CDK9	0.896
JUNB	0.938
TGFB3	0.938
HDAC1	0.958
SMAD4	0.958
TGFB1	0.958
PMEPA1	0.979

Supplementary Figure 1

1A

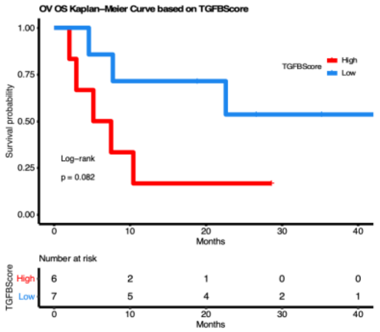

1B

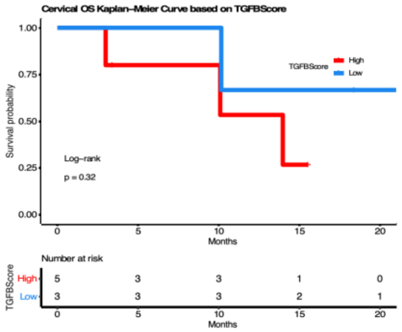

1C

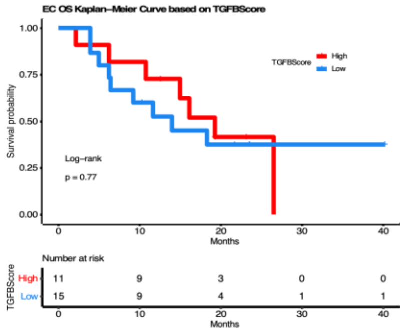

1D

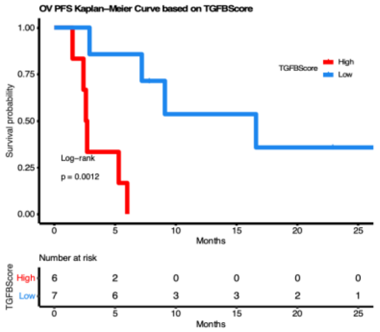

1E

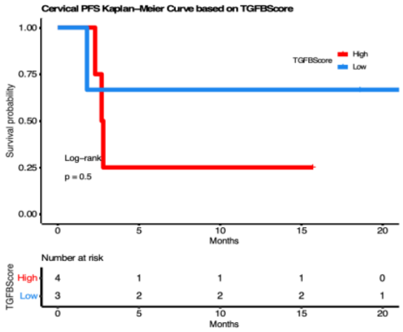

1F

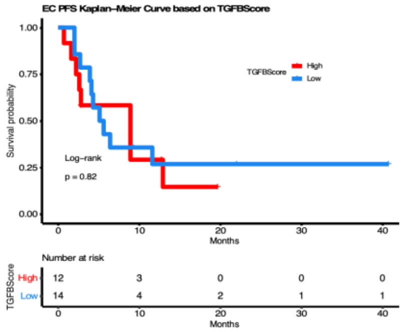

1G

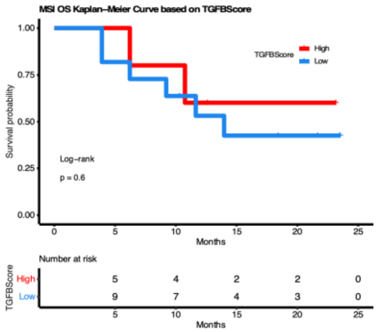

1H

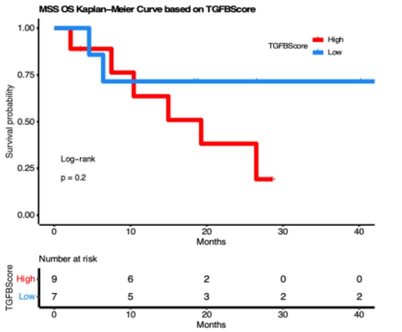

1K

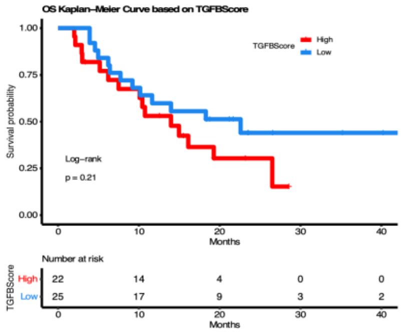

1I

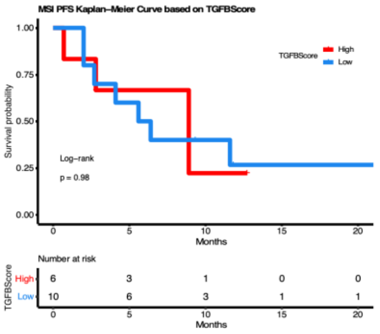

1J

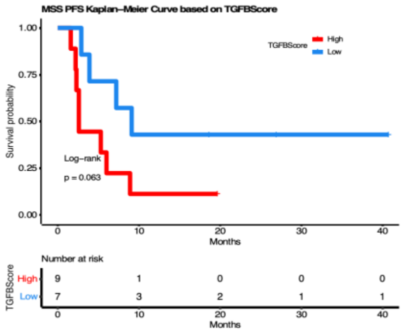

1L

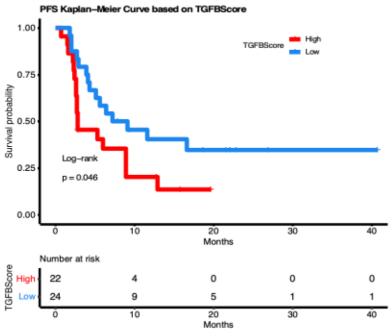

Supplementary Figure 2

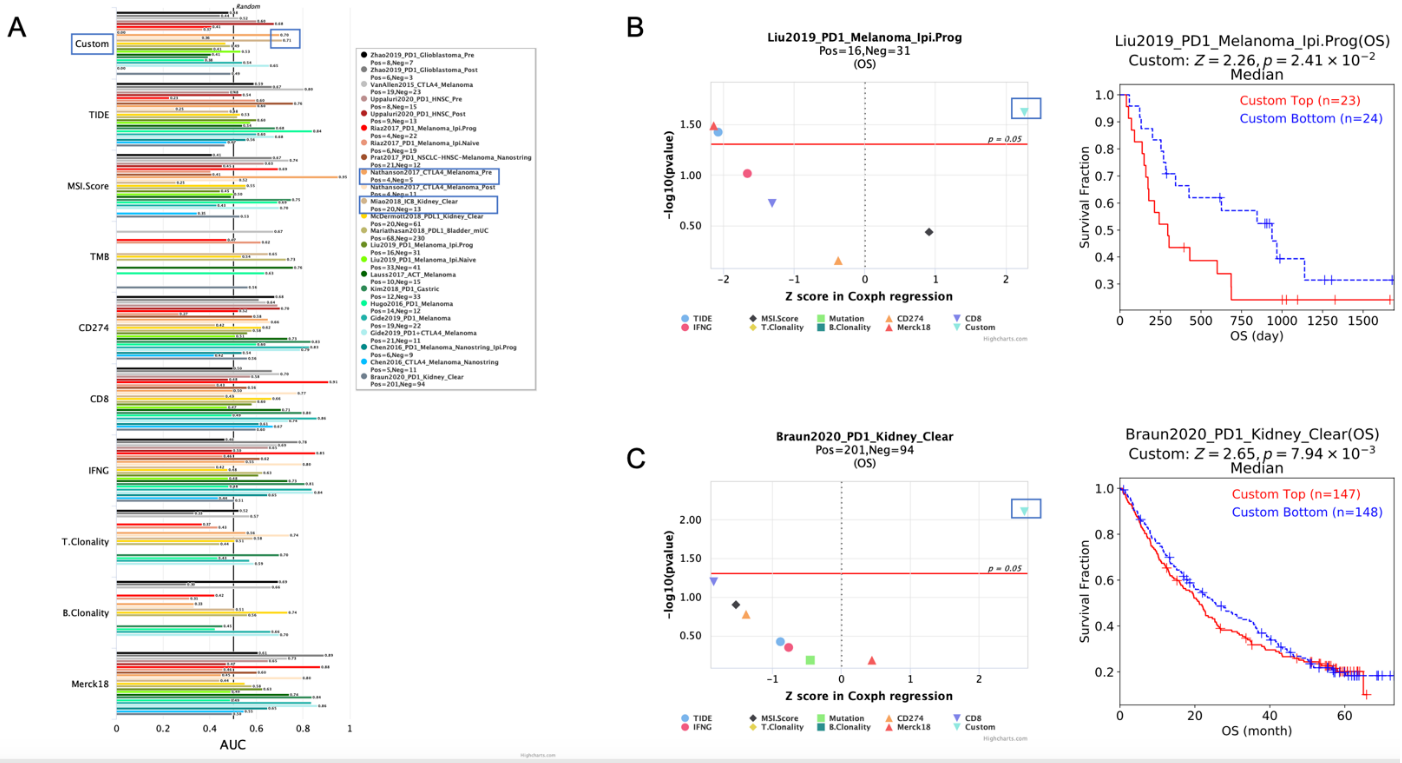

Supplementary Figure 3

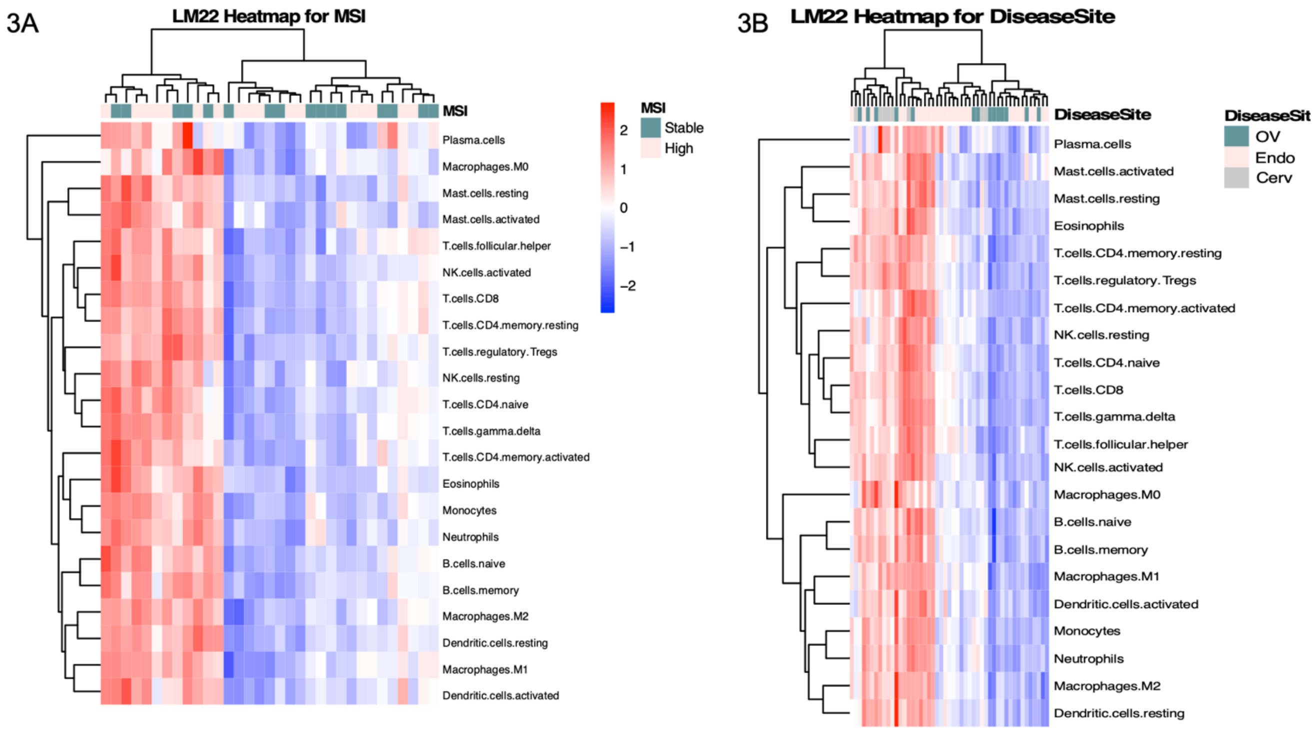

Supplemental Figure 1. Kaplan–Meier curves based on TGF-β score in individual cancer types and MSI status. Panel A, B, C, G, H: Kaplan–Meier curves of OS based on TGF-β score in ovarian, cervical, endometrial cancers, and in patients with MSI and MSS status; Panel D, E, F, I, J: Kaplan–Meier curves of PFS based on TGF-β score in ovarian, cervical, endometrial cancers, and in patients with MSI and MSS status. Panel K, L: Kaplan–Meier curves of OS and PFS based on TGF-β score in the entire cohort.

Supplemental Figure 2. TIDE validation of 6-gene TGF-β signature in multiple cancer datasets with immunotherapy. A: Our TGF-β score as annotated “Custom” panel at the top, showed a good predictive value (AUC > 0.7) in 2 studies (Nathanson

2017_CTLA4_Melanoma_Pre and Miao2018_ICB_Kidney_Clear, highlighted in blue squares). B: Significant predictive value based on Coxph regression with our TGF-β score (light blue triangle highlighted in the dark blue square) compared to other existing biomarkers in Liu2019_PD1_Melanoma_Ipi study, with corresponding Kaplan Meier curve based on our TGF-β score median cut-off in the same study. C: Significant predictive value based on Coxph regression with our TGF-β score (light blue triangle highlighted in the dark blue square) compared to other existing biomarkers in Braun2020_PD1_Kidney_Clear, with corresponding Kaplan Meier curve based on our TGF-β score median cut-off in the same study.

Supplemental Figure 3. Heatmap of all 22 immune cell abundance and status (LM22) in the entire cohort, sorted by MSI status (3A) and disease site (3B), scaled within each cell type.

Supplemental Table 1. Differentially expressed genes in ovarian and cervical cancer. There are 5 genes with known gene symbol overlapped between these 2 cancer types.

Supplemental Table 2. Canonical pathways curated based on differentially expressed genes using IPA.

Supplemental Table 3. Genes curated for TGF-β signaling and their association with response to immunotherapy.
